# ﻿Three new species of *Colletotrichum* (Glomerellales, Glomerellaceae) associated with walnut (*Juglansregia*) anthracnose from China

**DOI:** 10.3897/mycokeys.108.125382

**Published:** 2024-09-03

**Authors:** Yixuan Li, Lu Lin, Jing Cao, Mingxu Gan, Xinlei Fan

**Affiliations:** 1 State Key Laboratory of Efficient Production of Forest Resources, Beijing Forestry University, Beijing 100083, China; 2 Key Laboratory for Silviculture and Conservation of the Ministry of Education, Beijing Forestry University, Beijing 100083, China; 3 Ankang Forestry Technology Promotion Centre, Ankang, Shaanxi 725099, China; 4 Shangluo Forestry Extension Centre, Shangluo, Shaanxi 726199, China

**Keywords:** Glomerellaceae, novel species, systematics, taxonomy

## Abstract

*Colletotrichum* species are significant pathogens of various economic plant hosts worldwide. In this study, 45 *Colletotrichum* isolates were obtained from symptomatic walnut leaves of walnut anthracnose in Shaanxi and Sichuan Provinces. In conjunction with morphological evidence and multi-gene phylogenetic analyses of internal transcribed spacer (ITS), actin (*act), chitin synthase 1 (chs1), glyceraldehyde-3-phosphate dehydrogenase (gapdh*) and beta-tubulin (*tub2*) sequences support the introduction of three new species, namely *Colletotrichumcordae*, *C.guangyuanense* and *C.juglandium*. Five species of *Colletotrichum* were identified to be *C.fioriniae* of the *C.acutatum* species complex, *C.karsti* of the *C.boninense* species complex, *C.gloeosporioides*, *C.mengyinense* and *C.siamense* of the *C.gloeosporioides* species complex. The three new species are described and illustrated in this paper and compared with taxa in the *Colletotrichumgloeosporioides* species complex. The current results improve the understanding of *Colletotrichum* species causing walnut anthracnose in China.

## ﻿Introduction

Walnut (*Juglansregia* L.) is an economically significant woody nut and edible oil tree cultivated globally. It is widely grown across various regions in America, Asia, and Europe (Liu et al. 2021). According to the FAO statistics (http://www.fao.org/faostat, accessed on 20 March 2024), China is recognized as the world’s largest walnut producer, with over 390,000 hectares dedicated to walnut cultivation (Liu et al. 2021). Since 2017, China has consistently maintained its leading position in global walnut production (Da Lio et al. 2018; Li et al. 2023). Walnut plantations offer substantial economic, social, and ecological benefits (Nie et al. 2016). However, anthracnose induced by *Colletotrichum* species remains a major hurdle in walnut production worldwide, causing significant losses in productivity, including total crop failures (Wang et al. 2016). For example, *Colletotrichumaenigma*, *C.fructicola*, *C.gloeosporioides*, *C.liaoningense*, *C.siamense* and *C.sojae* were reported to cause anthracnose of walnut in Beijing Province (Li et al. 2023).

*Colletotrichum* (Glomerellaceae, Glomerellales, Sordariomycetes) is one of the most important and destructive plant pathogens worldwide (Dean et al. 2012). Traditionally, identifying *Colletotrichum* species based solely on morphological characteristics and host ranges has been challenging (Walker 1980). Consequently, systematic studies of *Colletotrichum* species complexes have underscored the importance of a multiphasic approach, integrating locus phylogeny with morphological, geographical, and ecological data to accurately characterize and identify *Colletotrichum* species (Cai et al. 2009; Damm et al. 2009, 2012a, b; Rojas et al. 2010; Liu et al. 2011; Weir et al. 2012; Marin-Felix et al. 2017; Jayawardena et al. 2020). The current taxonomy of the genus encompasses over 300 species, organized into 16 species complexes, with additional singletons (Marin-Felix et al. 2017; Liu et al. 2022; Talhinhas and Baroncelli 2023; Zapata et al. 2024).

Research on walnut anthracnose in China’s primary walnut-producing regions has identified fourteen *Colletotrichum* species associated with the disease (Zhang et al. 2023a). *Colletotrichumfructicola*, *C.gloeosporioides*, *C.siamense* and *C.viniferum* have been reported to be associated with walnut anthracnose in Shandong Province (Zhu et al. 2014; Wang et al. 2017, 2018; He et al. 2019). Moreover, *C.aenigma* has been implicated in Hebei Province, *C.fioriniae* in Guangxi Province, and *C.nymphaeae* in Gansu Province as pathogens of walnut anthracnose (Zhu et al. 2015; Wang et al. 2021; Ma et al. 2022). In Hubei Province, additional species including *C.fioriniae*, *C.gloeosporioides*, *C.godetiae*, *C.juglandis*, *C.kahawae*, and *C.nymphaeae* have been reported in association with the disease (Wei et al. 2022). *Colletotrichumgodetiae* has been identified as a cause of severe anthracnose in walnuts in Shaanxi and Yunnan Provinces. Additionally, in Beijing, a range of species including *C.aenigma*, *C.fructicola*, *C.gloeosporioides*, *C.juglandicola*, *C.liaoningense*, *C.peakense*, *C.siamense*, and *C.sojae* have been reported (Li et al. 2023; Wang et al. 2023; Zhang et al. 2023a). Based on previous studies of walnut anthracnose, it is generally accepted that *C.gloeosporioides* is the main pathogen of walnut anthracnose in China (Qu et al. 2011; Wang et al. 2016). Furthermore, recent studies have revealed a diverse array of *Colletotrichum* species associated with walnut anthracnose, such as *C.acutatum*, *C.aenigma*, *C.fioriniae*, *C.fructicola*, and *C.siamense*, among others, as noted by Li et al. (2023). Therefore, the exploration of pathogen diversity within walnut anthracnose continues to be an essential field of study. In this study we investigated the phylogenetic diversity of *Colletotrichum* species associated with walnut anthracnose in Shaanxi and Sichuan Provinces. We aimed to classify the isolates from this study based on phylogenetic analyses and morphological characteristics.

## ﻿Materials and methods

### ﻿Sample Collection and fungal isolation

**Figure 1.**
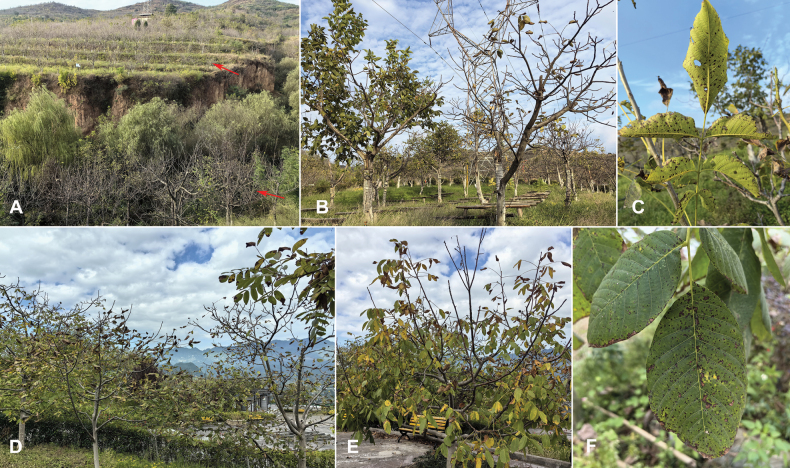
Disease symptoms on *Juglansregia* L. caused by *Colletotrichum* species (**A–F**). Red arrows point to symptoms of branches dieback caused by walnut anthracnose.

A total of 45 isolates were isolated from 25 walnut leaf samples with symptoms of anthracnose and collected in Shaanxi and Sichuan Provinces in China. The walnut anthracnose is characterized by small brown or black dry spots (Fig. 1). About 25 mm^2^ tissue fragments were taken from the margin of tissue lesions and the surfaces were sterilized with 75% ethanol for 30 s and 5% sodium hypochlorite for 60 s, rinsed in sterile distilled water for 60 s, and the samples were dried on aseptic filter paper (Gao et al. 2013; Liu et al. 2015). The sterilized sample was then placed in potato dextrose agar (PDA, 200 g potato, 20 g glucose, 20 g agar and 1 L distilled water) and cultured at 25 °C until mycelium grew from the sample. Then hyphae were picked out of the periphery of the colonies and inoculated onto Oatmeal Agar (OA, 30 g oatmeal, 15 g agar and 1 L distilled water) medium to promote the formation of spores. These leaf specimens are kept at the Museum of Beijing Forestry University (BJFC). The cultures are deposited in the China Forestry Culture Collection Centre (CFCC; http://www.cfcc-caf.org.cn/).

### ﻿Morphological analyses

Conidial structure and size were imaged with Leica stereo microscope (M205) (Leica Microsystems, Wetzlar, Germany). Conidia and other microstructures were randomly selected and observed by using Nikon Eclipse 80i microscope (Nikon Corporation, Tokyo, Japan) equipped with a Nikon digital sight DSRi-2 high-definition color camera with differential interference contrast (DIC). Fifty conidia were selected randomly to measure their lengths and widths. Colony morphology was observed on PDA and OA cultured at 25 °C. According to the color map of Rayner (1970) the color of the colony was described. The colony diameter was measured after 5 and 14 days.

### ﻿DNA extraction, PCR amplification and sequencing

Mycelium was collected from isolates grown on PDA agar and genomic DNA extraction was performed using the modified CTAB method (Doyle and Doyle 1990). First, the internal transcribed spacer (ITS) of all isolates was sequenced. The other genes were obtained from five nuclear gene regions: the glyceraldehyde-3-phosphate dehydrogenase gene (*gapdh*), chitin synthase 1 gene (*chs1*), actin gene (*act*), beta-tubulin gene (*tub2*) and histone H3 gene (*his3*) by using the primer pairs GDF1/GDR1, CHS-79F/CHS-345R, ACT-512F/ACT-783R, T1/Bt2b and CYLH3F/CYLH3R, respectively. The total volume of the PCR mixture is 20 µL, including 1 µL DNA template, 1 µL each 10 µM primer, 10 µL T5 Super PCR Mix and 7 µL sterile water. The gene fragments and amplification conditions used were in accordance with the details shown in Table 1 (Liu et al. 2022). The PCR products were electrophoresed in 1% agarose gel, and the DNA was sequenced by Sino Geno Max Biotechnology Company Limited (Beijing, China).

**Table 1.** Genes used in this study with PCR primers and optimal annealing temperature.

Locus	PCR Primers	PCR: Thermal Cycles: (Annealing Temp. in Bold)	Reference
ITS	ITS1/ITS4	(95 °C: 30 s, 51 °C: 30 s, 72 °C: 1 min) × 35 cycles	White et al. (1990)
*act*	ACT-512F/ACT-783R	(95 °C: 45 s, 55 °C: 45 s, 72 °C: 1 min) × 35 cycles	Carbone and Kohn (1999)
*chs1*	CHS-79F/CHS-345R	(95 °C: 30 s, 58 °C: 30 s, 72 °C: 1 min) × 35 cycles	Carbone and Kohn (1999)
*gapdh*	GDR1/GDF1	(95 °C: 30 s, 58 °C: 30 s, 72 °C: 1 min) × 35 cycles	Guerber et al. (2003)
*his3*	CYLH3F/CYLH3R	(95 °C: 30 s, 58 °C: 30 s, 72 °C: 1 min) × 35 cycles	Crous et al. (2004)
*tub2*	T1/Bt2b	(95 °C: 30 s, 55 °C: 30 s, 72 °C: 1 min) × 35 cycles	Glass and Donaldson (1995)

### ﻿Phylogenetic analyses

The resulting DNA sequences were combined with the sequences of reference strains from Genbank (Supplementary Suppl. material 1), and each single-gene dataset was aligned on MAFFT v. 6 separately (Katoh and Standley 2013), with both ends cut. Phylogenetic analysis of *C.acutatum* and *C.boninense* species complex was performed by combining six loci (ITS, *act*, *chs1*, *gapdh*, *his3* and *tub2*). Phylogenetic analysis of *C.gloeosporioides* species complex was performed by combining five loci (ITS, *act*, *chs1*, *gapdh*, and *tub2*). *Colletotrichumbambusicola* LC8469 *and C. orchidophilum* CBS 632.80 were used as the outgroup. Phylogenetic analyses of Maximum Likelihood (ML) and Bayesian Inference (BI) were performed. ML and BI analyses were computed using PhyML v. 3.0 (Guindon et al. 2010) and MrBayes v. 3.1.2 (Ronquist and Huelsenbeck 2003). For BI analysis, the best-fitting evolutionary model for each partitioned locus was estimated using the Markov Chain Monte Carlo algorithm in MrModelTest v. 2.3 (Posada and Crandall 1998). The system diagram is plotted in FigTree v. 1.4.3 (Rambaut and Drummond 2010) (http://tree.bio.ed.ac.uk/software/figtree) and edited in Adobe Illustrator 2019 (https://www.adobe.com/cn/products/illustrator.html). Sequence data were submitted to GenBank (https://www.ncbi.nlm.nih.gov) (Suppl. material 1).

## ﻿Results

### ﻿Phylogenetic analyses

Forty-five strains of *Colletotrichum*, isolated from leaves of *Juglansregia* L., were identified based on phylogenetic analyses of six loci. In the phylogenetic analysis of the *C.acutatum* species complex, a total of 2238 characters, including gaps, were identified (ITS: 549, *act*: 248, *chs1*: 282, *gapdh*: 267, *his3*: 390 and *tub2*: 502). Similarly, the phylogenetic analysis of the *C.boninense* species complex yielded a total of 2639 characters, including gaps (ITS: 592, *act*: 248, *chs1*: 300, *gapdh*: 321, *his3*: 410 and *tub2*: 768). An additional analysis of the *C.gloeosporioides* species complex resulted in 2294 characters, including gaps (ITS: 575, *act*: 323, *chs1*: 300, *gapdh*: 348 and *tub2*: 748). The GTR+I+G model was proposed for ITS, *act* and *gapdh*, and the HKY+I+G model was proposed for *chs1*, *his3* and *tub2* (Ronquist and Huelsenbeck 2003). The best-fit models used the statistics of ML trees are shown in Suppl. material 2. Both Maximum Likelihood (ML) and Bayesian Inference (BI) methods were employed in these analyses. The topology of Bayesian analysis of cascading datasets is almost the same as ML consistency tree.

**Figure 2.**
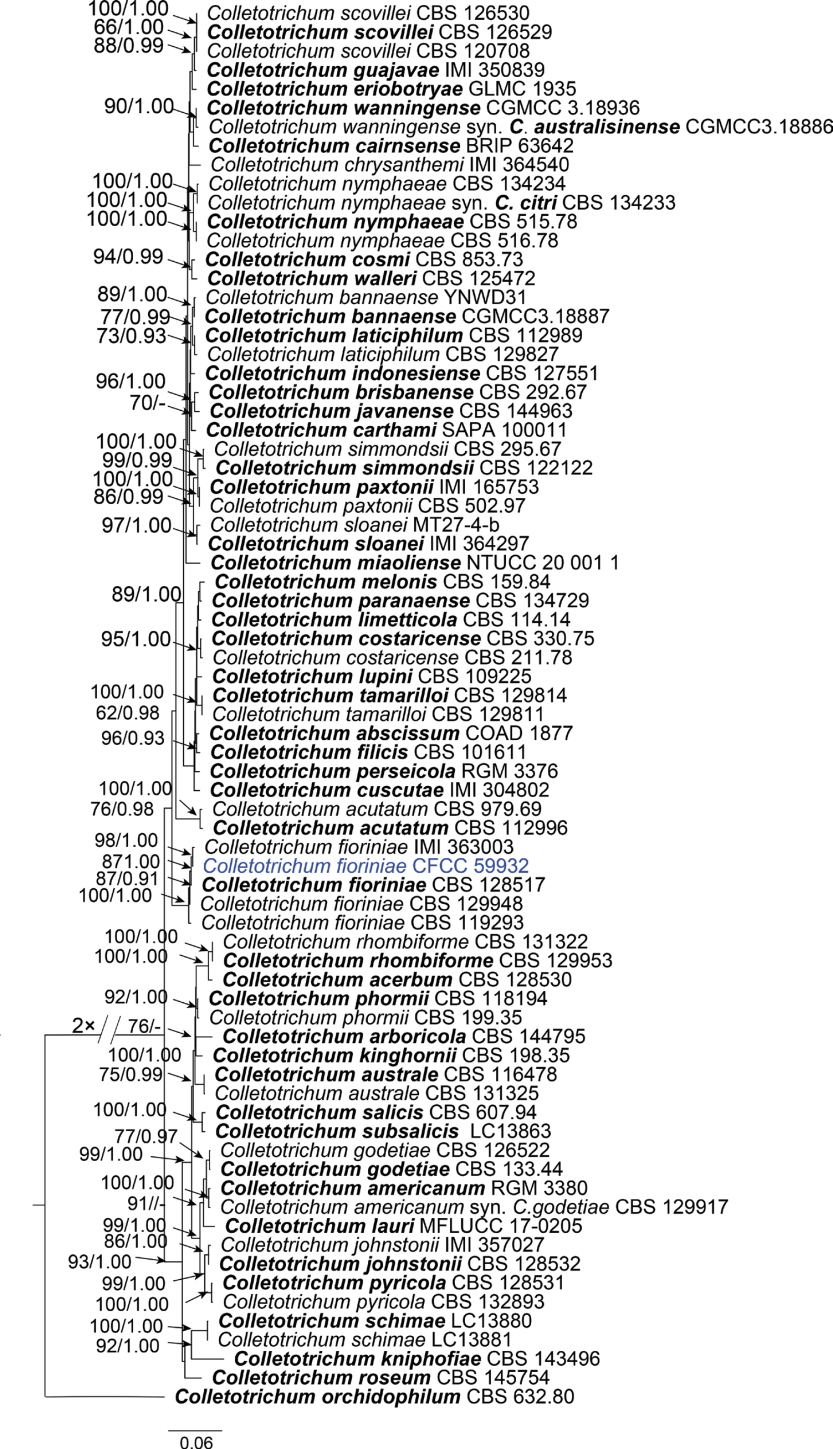
Phylogram of *Colletotrichumacutatum* complex species based on Maximum Likelihood (ML) analysis of the dataset of combined ITS, *gapdh*, *act*, *tub2*, *chs1* and *his3* genes. ML bootstrap support values above 60% and Bayesian posterior probability above 0.90 are shown near nodes. Ex-type cultures are in bold. Isolates obtained in this study are highlighted with blue colors.

**Figure 3.**
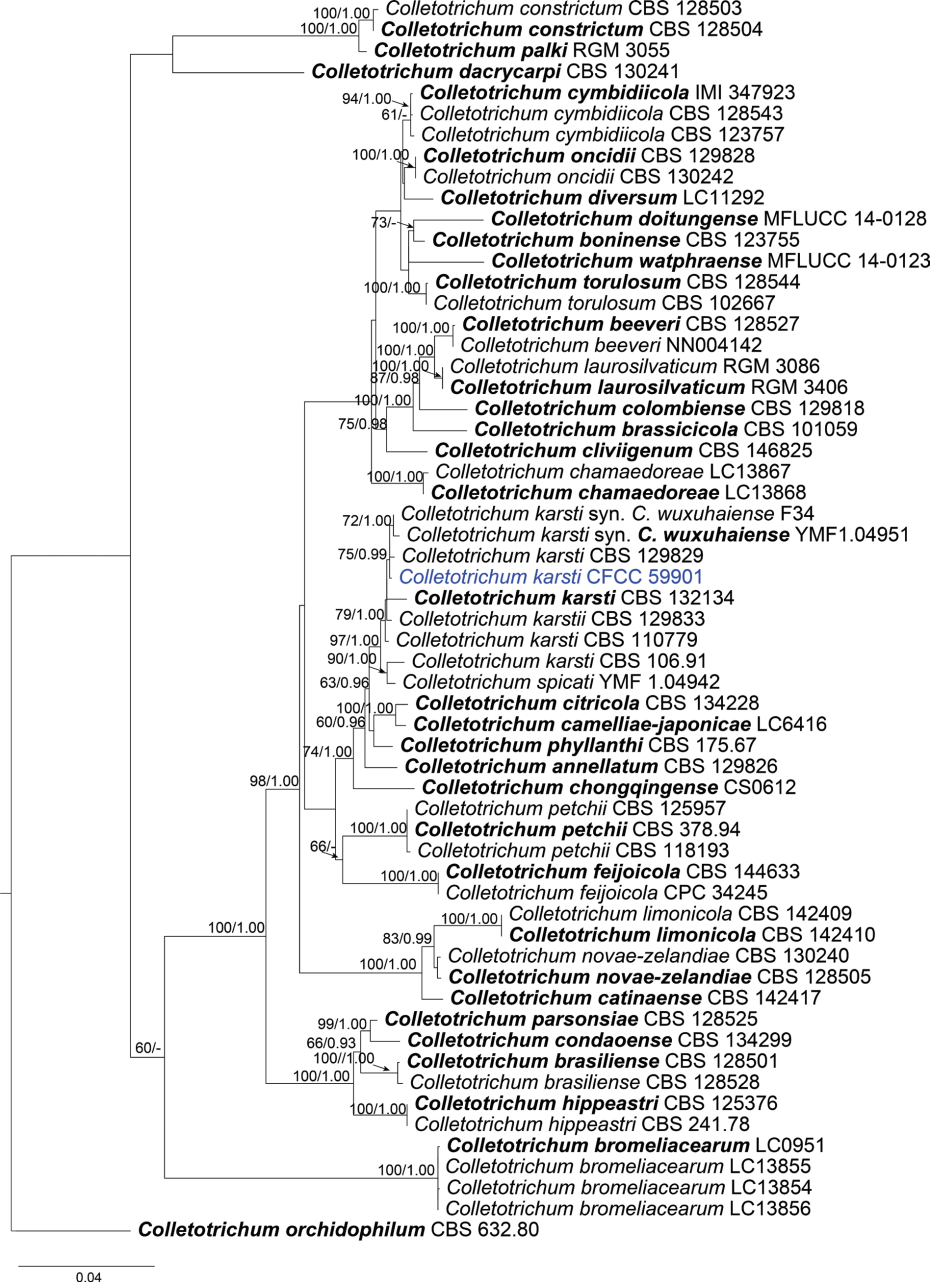
Phylogram of *Colletotrichumboninense* complex species based on Maximum Likelihood (ML) analysis of the dataset of combined ITS, *gapdh*, *act*, *tub2*, *chs1* and *his3* genes. ML bootstrap support values above 60% and Bayesian posterior probability above 0.90 are shown near nodes. Ex-type cultures are in bold. Isolates obtained in this study are highlighted with blue colors.

**Figure 4.**
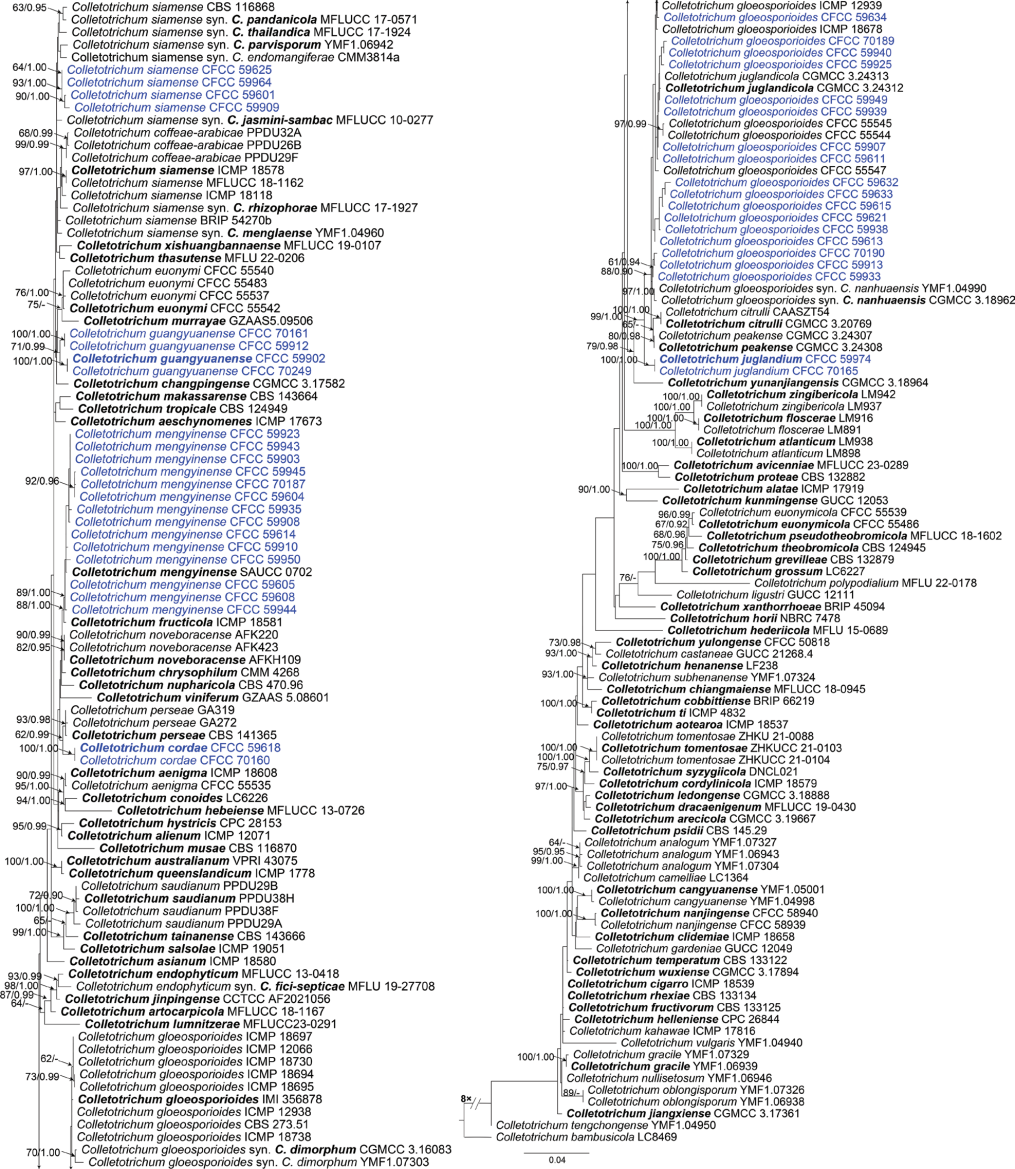
Phylogram of *Colletotrichumgloeosporioides* complex species based on Maximum Likelihood (ML) analysis of the dataset of combined ITS, *gapdh*, *act*, *tub2* and *chs1* genes. ML bootstrap support values above 60% and Bayesian posterior probability above 0.90 are shown near nodes. Ex-type cultures are in bold. Isolates obtained in this study are highlighted with blue colors.

The phylogenetic tree showed 45 isolates across three species complexes: the *C.acutatum* species complex with a single isolate (Fig. 2), the *C.boninense* species complex with a single isolate (Fig. 3), and the *C.gloeosporioides* species complex with 43 isolates (Fig. 4). In the *C.acutatum* species complex, one isolate clustered with four reference isolates of *C.fioriniae*. In the *C.boninense* species complex, one isolate clustered with seven reference isolates of *C.karsti*. In the *C.gloeosporioides* species complex, 14 isolates clustered together with *C.mengyinense*, 17 isolates clustered with *C.gloeosporioides* and four isolates clustered with *C.siamense*, eight isolates formed three separate clades with high support (Fig. 4).

### ﻿Taxonomy

#### ﻿ Colletotrichum cordae

Taxon classificationFungiGlomerellalesGlomerellaceae

Y.X. Li & X.L. Fan sp. nov.

F2FB6293-7524-5424-942D-35A322C964FF

 852127

Fig. 5

##### Etymology.

Named after Corda who established the genus *Colletotrichum*.

##### Typification.

China, Sichuan Province, Guangyuan City, Chaotian District, Longmen Valley Leisure Villa, 32°39'08"N, 105°55'11"E, from leaf of *Juglansregia* L., 10 Oct. 2023, Y.X. Li, L. Lin & X.L. Fan (holotype BJFC-S2250, ex-holotype culture CFCC 59618).

**Figure 5.**
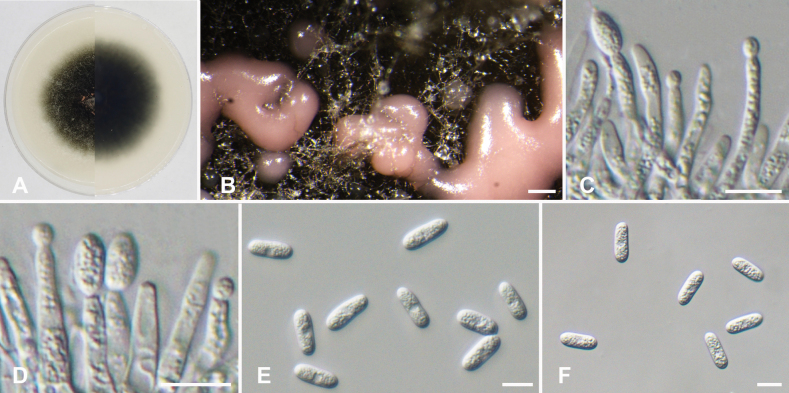
*Colletotrichumcordae* (ex-holotype culture CFCC 59618) **A** colonies on OA media above and below after 5 days at 25 °C **B** conidiomata on OA **C, D** conidiophores and conidia **E, F** conidia. Scale bars: 200 µm (**B**); 10 µm (**C–F**).

##### Description.

***Sexual morph*** not observed. ***Asexual morph*** developed on OA. ***Conidiomata*** acervular, color ranged from peach to light brown. ***Appressoria*** and ***Setae*** not observed on OA. ***Conidiophores*** directly formed on hyphae, usually degenerated into conidiogenous cells. ***Conidiophores*** hyaline, unbranched, approximately cylindrical, 16.1–28.2 × 2.5–4.5, mean ± SD = 21.0 ± 2.9 × 3.5 ± 0.5 µm, n = 50. ***Conidiogenous cells*** transparent, cylindrical, formed at the end or side of the hyphae. ***Conidia*** straight, hyaline, cylindrical, obtuse at the base, rounded at the apex, with smooth walls and granular contents, 11.8–17.7 × 4.5–6.5 µm, mean ± SD = 14.5 ± 1.1 × 5.5 ± 0.5 µm, L/W radio = 2.6, n = 50.

##### Cultural characteristics.

Colonies on OA initially white, rapidly growing to 4 cm after 3 d at 25 °C, and completely covering a 6-centimeter Petri dish after 7 d. The aerial mycelium white or gray, with a flocculent cotton-like appearance, edge white, center iron gray.

##### Additional material examined.

China. Sichuan Province, Guangyuan City, Chaotian District, Longmen Valley Leisure Villa, 32°39'08"N, 105°55'11"E, from leaf of *Juglansregia* L., 10 Oct. 2023, Y.X. Li, L. Lin & X.L. Fan (BJFC-S2251, living culture CFCC 70160).

##### Notes.

Two strains of *Colletotrichumcordae* constitute a distinct clade within the *C.gloeosporioides* species complex, as revealed by multi-locus phylogenetic analysis. *Colletotrichumcordae* is phylogenetically near to *C.perseae* CBS 141365, but differs by 17 nucleotide differences in concatenated alignment (7/573 in ITS, 5/281 in *act*, 1/269 in *chs1*, 1/317 in *gapdh*, and 3/452 in *tub2*) (Sharma et al. 2017). Morphologically, *C.cordae* can be differentiated from *C.perseae* by the presence of conidia with obtuse or rounded apices.

#### ﻿ Colletotrichum fioriniae

Taxon classificationFungiGlomerellalesGlomerellaceae

(Marcelino & Gouli) R.G. Shivas & Y.P. Tan, Fungal Divers. 39: 117, 2009

FB7FD827-6948-528D-87F2-3993B03D967B

##### Material examined.

China. Shaanxi Province, Shangluo City, Lonan County, Red kernel walnut base, 34°03'10"N, 110°14'11'′E, from leaf of *Juglansregia* L., 14 Oct. 2023, Y.X. Li, L. Lin & X.L. Fan (BJFC-S2253, living culture CFCC 59932).

##### Notes.

*Colletotrichumfioriniae*, a worldwide fungus with a wide range of host, is associated with walnut anthracnose disease (Shivas and Tan 2009; Zhu et al. 2015). Relative to other species within the *Colletotrichum* genus, *C.fioriniae* is generally considered less prevalent and less virulent, as noted by Talhinhas and Baroncelli (2021). In our research, the strain CFCC 59932 clusters robustly with *C.fioriniae* on both Maximum Likelihood (ML) and Bayesian Inference (BI) phylogenetic trees, indicating strong statistical support (ML/BI = 100/1).

#### ﻿ Colletotrichum gloeosporioides

Taxon classificationFungiGlomerellalesGlomerellaceae

(Penz.) Penz. & Sacc., Atti Reale Ist. Veneto Sci. Lett. Arti., ser. 6, 2: 670. 1884

A3ADA95E-B723-545D-9E3C-9CDB3B4B517D

##### Materials examined.

China. • Sichuan Province, Guangyuan City, Chaotian District, Walnut Cultural Square, 32°40'58"N, 106°02'08"E, from leaf of *Juglansregia* L., 10 Oct. 2023, Y.X. Li, L. Lin & X.L. Fan (BJFC-S2239, living culture CFCC 59613; BJFC-S2240, living culture CFCC 59621; BJFC-S2242, living culture CFCC 59634); • Chaotian District, Mianguang Expressway, 32°40'50"N, 105°59'19"E, from leaf of *Juglansregia* L., 10 Oct. 2023, Y.X. Li, L. Lin & X.L. Fan (BJFC-S2246, living culture CFCC 59611; BJFC-S2247, living culture CFCC 59615; BJFC-S2244, living culture CFCC 59632); • Chaotian District, Zhongzi Town, 32°41'34"N, 106°02'23"E, from leaf of *Juglansregia* L, 10 Oct. 2023, Y.X. Li, L. Lin & X.L. Fan (BJFC-S2230, living culture CFCC 59633; BJFC-S2228, living culture CFCC 59907; BJFC-S2252, living culture CFCC 59913; BJFC-S2254, living culture CFCC 59933). China. • Shaanxi Province, Shangluo City, Danfeng County, Dihua Ancient Town, 33°44'23"N, 110°12'07"E, from leaf of *Juglansregia* L., 13 Oct. 2023, Y.X. Li, L. Lin & X.L. Fan (BJFC-S2257, living culture CFCC 59938; BJFC-S2258, living culture 59939); • Danfeng County, Walnut Theme Park, 33°44'33"N, 110°11'55"E, from leaf of *Juglansregia* L., 13 Oct. 2023, Y.X. Li, L. Lin & X.L. Fan (BJFC-S2263, living culture CFCC 59925; BJFC-S2264, living culture CFCC 70189; BJFC-S2259, living culture CFCC 59940; BJFC-S2267, living culture CFCC 59949; BJFC-S2261, living culture CFCC 70190).

##### Notes.

*Colletotrichumgloeosporioides* was originally described as *Vermiculariagloeosporioides* and collected from *Citrus* sp. in Italy. The current name *Colletotrichumgloeosporioides* was proposed by Penzig (1882). *Colletotrichumgloeosporioides* is a worldwide fungus that inhabits a wide range of host plants. In our study, 17 strains and three species (i.e., *C.citrulli*, *C.juglandicola* and *C.peakense*) were robustly grouped with *C.gloeosporioides*, supported by high Maximum Likelihood (ML) and Bayesian Inference (BI) confidence values (ML/BI = 88/0.90). Zhang et al. (2023b) reduced *C.dimorphum* and *C.nanhuaense* as synonyms of *C.gloeosporioides*. Further research is needed to confirm the taxonomic status of *C.citrulli*, *C.juglandicola* and *C.peakense*. The morphology of the strains in our study closely resembles the type specimen of *C.gloeosporioides*, as described by Cannon et al. (2008). Thus, we propose the identification of our strains as *C.gloeosporioides*, based on both morphological characteristics and phylogenetic analyses. The result proves that walnut anthracnose has been attributed to *C.gloeosporioides* (Wang et al. 2020a; Mu et al. 2021; Yang et al. 2021; Li et al. 2023).

#### ﻿ Colletotrichum guangyuanense

Taxon classificationFungiGlomerellalesGlomerellaceae

Y.X. Li & X.L. Fan sp. nov.

2A654579-9F22-5F0B-97E3-74AA4912115F

 852126

Fig. 6

##### Etymology.

Named after the location where the fungal was first collected, which is Guangyuan City.

##### Typification.

China, Sichuan Province, Guangyuan City, Lizhou District, Shuiwo Village, 32°23'55"N, 105°39'24"E, from leaf of *Juglansregia* L., 11 Oct. 2023, Y.X. Li, L. Lin & X.L. Fan (holotype BJFC-S2225, ex-holotype culture CFCC 59902).

**Figure 6.**
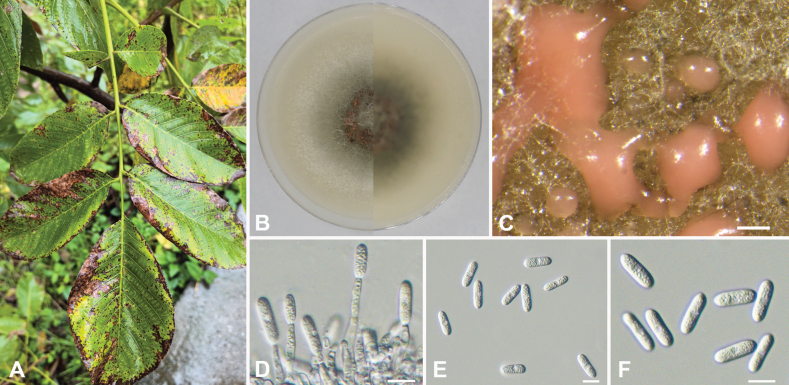
*Colletotrichumguangyuanense* (ex-holotype culture CFCC 59902) **A** symptom caused by *Colletotrichumguangyuanense***B** colonies on OA media above and below after 5 days at 25 °C **C** conidiomata on OA **D** conidiophores and conidia **E, F** conidia. Scale bars: 200 µm (**C**); 10 µm (**D–F**).

##### Description.

***Sexual morph*** not observed. ***Asexual morph*** developed on OA. ***Conidiomata*** acervular, color ranged from peach to light brown. ***Appressoria*** and ***Setae*** not observed on OA. ***Conidiophores*** hyaline, unbranched, approximately cylindrical, 22.2–35.1 × 3.2–5.3, mean ± SD = 27.4 ± 3.1 × 3.8 ± 0.5 µm, n = 50. ***Conidiogenous cells*** transparent, cylindrical, formed at the end or side of the hyphae. ***Conidia*** straight, hyaline, cylindrical, obtuse at the base, rounded at the apex, with smooth walls and granular contents, 9.7–17.7 × 3.9–6.9 µm, mean ± SD = 14.7 ± 1.9 × 5.5 ± 0.6 µm, L/W radio = 2.7, n = 100.

##### Culture characteristics.

Colonies on OA initially white, rapidly growing to 5 cm after 3 d at 25 °C, and completely covering a 6 cm Petri dish after 7 d. The aerial mycelium white or gray, with a flocculent cotton like appearance, edge white, center pale greenish grey.

##### Additional materials examined.

China. • Sichuan Province, Guangyuan City, Lizhou District, Shuiwo Village, 32°23'31"N, 105°39'22"E, from leaf of *Juglansregia* L., 11 Oct. 2023, Y.X. Li, L. Lin & X.L. Fan (BJFC-S2226, living culture CFCC 70249). China. • Sichuan Province, Guangyuan City, Chaotian District, Longmen Valley Leisure Villa, 32°39'08"N, 105°58'17"E, from leaf of *Juglansregia* L., 10 Oct. 2023, Y.X. Li, L. Lin & X.L. Fan (BJFC-S2248, living culture CFCC 59912); • Chaotian District, Longmen Valley Leisure Villa, 32°39'11"N, 105°55'26"E, from leaf of *Juglansregia* L., 10 Oct. 2023, Y.X. Li, L. Lin & X.L. Fan (BJFC-S2249, living culture CFCC 70161).

##### Notes.

In phylogenetic analyses, *Colletotrichumguangyuanense* forms a distinct clade within the *C.gloeosporioides* species complex, closely related to *C.changpingense*. Genetic differences between *C.guangyuanense* and the type strain of *C.changpingense* are observed at several loci: 4 bp in the ITS region, 3 bp in the *act* gene, 8 bp in the *chs1* gene, 6 bp in the *gapdh* gene, and 1 bp in the *tub2* gene (Jayawardena et al. 2016a). Morphologically, *C.guangyuanense* is distinguishable from *C.changpingense* by the absence of a distinct opaque region in the center of the conidia.

#### ﻿ Colletotrichum juglandium

Taxon classificationFungiGlomerellalesGlomerellaceae

Y.X. Li & X.L. Fan sp. nov.

922AE37C-8E8D-52FC-AC1C-F69BFE928562

 852125

Fig. 7

##### Etymology.

Named after the host genus on which it was collected, *Juglansregia* L.

##### Typification.

China. Sichuan Province, Guangyuan City, Chaotian District, Mianguang Expressway, 32°40'50"N, 105°59'19"E, from leaf of *Juglansregia* L., 10 Oct. 2023, Y.X. Li, L. Lin & X.L. Fan (holotype BJFC-S2243, ex-holotype culture CFCC 59974).

**Figure 7.**
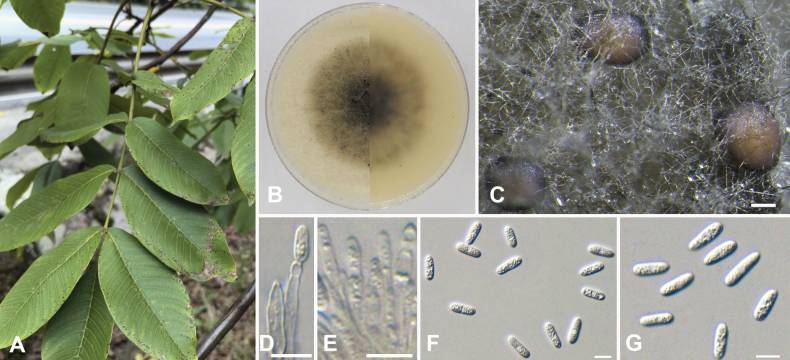
*Colletotrichumjuglandium* (ex-holotype culture CFCC 59974) **A** symptoms caused by *Colletotrichumjuglandium***B** colonies on OA media above and below after 5 days at 25 °C **C** conidiomata **D, E** conidiophores and conidia **F, G** conidia. Scale bars: 200 µm (**C**); 10 µm (**D–G**).

##### Description.

***Sexual morph*** not observed. ***Asexual morph*** on OA. ***Conidiomata*** acervular, color ranged from peach to light brown. ***Appressoria*** and ***Setae*** not observed on OA. ***Conidiophores*** hyaline, unbranched, approximately cylindrical, 16.0–27.6 × 2.2–4.7, mean ± SD = 20.1 ± 3.2 × 3.2 ± 0.6 µm, n = 30. ***Conidiogenous cells*** transparent, cylindrical, formed at the end or side of the hyphae. ***Conidia*** straight, hyaline, cylindrical, obtuse at the base, rounded at the apex, with smooth walls and granular contents, 13.2–22.4 × 4.4–6.3 µm, mean ± SD = 16.8 ± 1.8 × 5.4 ± 0.4 µm, L/W radio = 3.1, n = 50.

##### Culture characteristics.

Colonies on OA initially white, rapidly growing to 5 cm after 3 d at 25 °C, and completely covering a Petri dish after 7 days. The aerial mycelium white or gray, with a flocculent cotton like, edge white, center mouse grey.

##### Additional material examined.

China. Sichuan Province, Guangyuan City, Chaotian District, Mianguang Expressway, 32°40'36"N, 105°59'26"E, from leaf of *Juglansregia* L., 10 Oct. 2023, Y.X. Li, L. Lin & X.L. Fan (BJFC-S2245, living culture CFCC 70165).

##### Notes.

*Colletotrichumjuglandium* form a solitary clade the *C.gloeosporioides* species complex (Fig. 3). *Colletotrichumjuglandium* is closely related to *C.citrulli*, *C.gloeosporioides*, *C.juglandicola* and *C.peakense* (Zhang et al. 2023a). Sequence identity comparisons reveal that *C.juglandium*CFCC 59974 differs from other species at various loci: *C.citrulli* CGMCC3.20769 (3/544 in ITS, 0/244 in *act*, 0/228 in *chs1*, 10/297 in *gapdh*, and 0/324 in *tub2*), *C.gloeosporioides* IMI 356878 (2/544 in ITS, 0/289 in *act*, 0/236 in *chs1*, 18/341 in *gapdh*, and 0/324 in *tub2*), *C.juglandicola* CGMCC 3.24312 (2/544 in ITS, 0/279 in *act*, 1/249 in *chs1*, 18/341 in *gapdh*, and 1/324 in *tub2*), and *C.peakense* CGMCC 3.24308 (2/544 in ITS, 0/279 in *act*, 1/249 in *chs1*, 13/341 in *gapdh*, and 1/324 in *tub2*) (Guo et al. 2022; Yu et al. 2022; Zhang et al. 2023a). Morphologically, *C.juglandium* differs from *C.gloeosporioides* by having narrower conidia (L/W ratio: 3.1 vs. 2.6) and differs from *C.juglandicola* and *C.peakense* by having wider conidia (L/W ratio: 3.1 vs. 3.3).

#### ﻿ Colletotrichum karsti

Taxon classificationFungiGlomerellalesGlomerellaceae

Y.L. Yang, Z.Y. Liu, K.D. Hyde & L. Cai, Cryptog. Mycol. 32: 241. 2011

39216FDC-8F10-5D0B-9131-701F5644AC45

##### Material examined.

China. Sichuan Province, Guangyuan City, Lizhou District, Shuiwo Village, 32°23'38"N, 105°39'22"E, from leaf of *Juglansregia* L., 11 Oct. 2023, Y.X. Li, L. Lin & X.L. Fan (BJFC-S2224, living culture CFCC 59901).

##### Notes.

*Colletotrichumkarsti* was identified as a pathogen on *Vanda* species, causing ellipsoid lesions on leaves, and was also found as an endophyte in roots in Guizhou Province, China. This species is recognized as the most prevalent and geographically widespread within the *C.boninense* species complex, with a broad host range (Yang et al. 2011; Damm et al. 2012a; Jayawardena et al. 2016b). Zhang et al. (2022) synonymized *C.wuxuhaiense* with *C.karsti*. In this study, the strain CFCC 59901 is confirmed to be *C.karsti* based on morphological characteristics and DNA sequence data. Furthermore, this represents a new host record for *C.karsti* on walnut trees.

#### ﻿ Colletotrichum mengyinense

Taxon classificationFungiGlomerellalesGlomerellaceae

T.C. Mu, J.W. Xia, X.G. Zhang & Z. Li, MycoKeys 85: 66 (2021)

1BF9EECA-1541-5E88-90BF-B0CE7F243B17

##### Materials examined.

China. • Sichuan Province, Guangyuan City, Chaotian District, Walnut Cultural Square, 32°40'58"N, 106°02'08"E, from leaf of *Juglansregia* L., 10 Oct. 2023, Y.X. Li, L. Lin & X.L. Fan (BJFC-S2241, living culture CFCC 59604; BJFC-S2236, living culture CFCC 59605; BJFC-S2237, living culture CFCC 59608; BJFC-S2238, living culture CFCC 59910); • Chaotian District, Cypress Bridge, 32°41'16"N, 106°02'22"E, from leaf of *Juglansregia* L., 10 Oct. 2023, Y.X. Li, L. Lin & X.L. Fan (BJFC-S2232, living culture CFCC 59614); • Chaotian District, Zhongzi Town, 32°41'34"N, 106°02'23"E, from leaf of *Juglansregia* L., 10 Oct. 2023,Y.X. Li, L. Lin & X.L. Fan (BJFC-S2229, living culture CFCC 59908); • Lizhou District, Tulongzi, 32°30'36"N, 105°36'51"E, from leaf of *Juglansregia* L., 11 Oct. 2023, Y.X. Li, L. Lin & X.L. Fan (BJFC-S2227, living culture CFCC 59903). China. • Shaanxi Province, Shangluo City, Danfeng County, Walnut Theme Park, 33°44'33"N, 110°11'55"E, from leaf of *Juglansregia* L., 13 Oct. 2023, Y.X. Li, L. Lin & X.L. Fan (BJFC-S2260, living culture CFCC 59923; BJFC-S2262, living culture CFCC 59943; BJFC-S2265, living culture CFCC 59944; BJFC-S2266, living culture CFCC 59945; BJFC-S2268, living culture CFCC 59950); • Shangzhou District, United Village, 33°52'03"N, 109°51'01"E, from leaf of *Juglansregia* L., 14 Oct. 2023, Y.X. Li, L. Lin & X.L. Fan (BJFC-S2256, living culture CFCC 59935; BJFC-S2255, living culture CFCC 70187).

##### Notes.

*Colletotrichummengyinense* was isolated originally on diseased leaves of *Rosachinensis* (Mu et al. 2021). Additionally, the current 14 isolates are morphologically not significantly different from *C.mengyinense* and aggregated together with *C.mengyinense* with high support (ML/BI = 89/1.00) on the phylogenetic tree. Therefore, they are identified as *Colletotrichummengyinense*.

#### ﻿ Colletotrichum siamense

Taxon classificationFungiGlomerellalesGlomerellaceae

Prihastuti, L. Cai & K.D. Hyde, Fungal Divers. 39: 98 (2009)

EE1B963D-283B-5E18-A125-5CB4A340083F

##### Materials examined.

China. • Sichuan Province, Guangyuan City, Chaotian District, Zhongzi Town, 32°41'05"N, 106°02'08"E, from leaf of *Juglansregia* L., 10 Oct. 2023, Y.X. Li, L. Lin & X.L. Fan (BJFC-S2231, living culture CFCC 59601; BJFC-S2233, living culture CFCC 59625; BJFC-S2235, living culture CFCC 59964); • 32°41'34"N, 106°02'23"E, from leaf of *Juglansregia* L., 10 Oct. 2023, Y.X. Li, L. Lin & X.L. Fan (holotype BJFC-S2234, living culture CFCC 59909)

##### Notes.

*Colletotrichumsiamense* was first described as a species in association with *Coffeaarabica* by Prihastuti et al. (2009). The broader concept of *C.siamensesensu lato* has been a subject of considerable debate, as noted by Weir et al. (2012) and Sharma et al. (2015), due to the application of the Genealogical Concordance Phylogenetic Species Recognition (GCPSR) approach. Liu et al. (2016) concluded that *C.siamense* is a single species, not a species complex. Based on phylogenetic evidence, Zhang et al. (2022) proposed that *C.menglaense*, *C.pandanicola*, and *C.parvisporum* are synonyms of *C.siamense*. More recent studies have further synonymized the closely related species *C.rhizophorae* and *C.thailandica* with *C.siamense*, considering morphological characteristics, phylogenetic analyses, and GCPSR (Aumentado et al. 2024). In this study, *C.siamense* was isolated from walnut leaf spots affected by walnut anthracnose. The four isolates our study examined while forming a distinct lineage were found within the *C.siamense* clade in our phylogenetic analysis. Moreover, our isolate (CFCC 59909) is similar to the holotype of *C.siamense* (ICMP 18578). While the conidia of our isolate (CFCC 59909) are wider than strain ICMP 18578 (12.3–14.5 × 4.9–6.4 vs. 7–18.3 × 3–4.3 μm) (Prihastuti et al. 2009). Based on this evidence, we identify our isolate as *C.siamense*.

## ﻿Discussion

The genus *Colletotrichum* comprises significant plant pathogens that impact many economically important crops globally. Despite notable advancements in the taxonomy of *Colletotrichum*, ongoing debates regarding its taxonomic relationships warrant further research (Cannon et al. 2008, 2012; Cai et al. 2009; Liu et al. 2013, 2016). This study focused on walnut anthracnose in Sichuan and Shaanxi Provinces of China, conducting phylogenetic analyses using DNA sequence data. Five known species (*C.fioriniae*, *C.gloeosporioides*, *C.karsti*, *C.mengyinense* and *C.siamense*) and three new species (*C.cordae*, *C.guangyuanense* and *C.juglandium*) associated with walnut anthracnose were identified in the current study.

The genus *Colletotrichum* exhibits considerable species diversity in infections of walnut hosts. In China, a variety of *Colletotrichum* species have been reported on walnut, including those from the *C.acutatum* species complex (e.g., *C.acutatum*, *C.fioriniae*, *C.godetiae*, *C.juglandicola*, *C.juglandis*, *C.nymphaeae*), the *C.gloeosporioides* species complex (e.g., *C.aenigma*, *C.fructicola*, *C.gloeosporioides*, *C.kahawae*, *C.mengyinense*, *C.peakense*, *C.siamense*, *C.viniferum*), the *C.magnum* species complex (e.g., *C.liaoningense*), and the *C.orchidearum* species complex (e.g., *C.sojae*). This list is based on numerous studies conducted over the years (Simmonds 1966; Alvarez 1976; Gorter 1977; Pennycook 1989; Liu et al. 1995; Chen 2003; Gadgil et al. 2005; Juhásová et al. 2005; Sreenivasaprasad and Talhinhas 2005; Kobayashi 2007; Qu et al. 2011; Damm et al. 2012b; Zhu et al. 2014, 2015; Wang et al. 2017, 2018; Da Lio et al. 2018; He et al. 2019; Savian et al. 2019; Wang et al. 2020b, 2021; Luongo et al. 2022; Ma et al. 2022; Wei et al. 2022; Li et al. 2023; Wang et al. 2023; Zhang et al. 2023a). Pathogenicity tests have demonstrated that numerous *Colletotrichum* species are responsible for anthracnose disease on walnut fruits and leaves, as Li et al. (2023) and Zhang et al. (2023a) reported. Wang et al. (2020a) revealed that the virulence of the pathogen of walnut anthracnose to walnut fruits was different. In this study, the pathogenicity of two established species, *C.mengyinense* and *C.karsti*, and three newly identified species, requires further investigation.

In the current study, five species (i.e., *C.citrulli*, *C.dimorphum*, *C.juglandicola*, *C.nanhuaense* and *C.peakense*) along with 17 isolates form part of the *C.gloeosporioides* clade in both Maximum Likelihood (ML) and Bayesian Inference (BI) phylogenetic trees. However, these species and isolates were unable to form clear branches in the phylogenetic tree. They also have overlapping morphological characters (Guo et al. 2022; Yu et al. 2022; Zhang et al. 2023a). Consequently, the 17 isolates in question have been identified as *C.gloeosporioides*. Liu et al. (2022) demonstrated that the boundaries between *C.gloeosporioides* and its closely related species are unclear. Zhang et al. (2023b) synonymized *C.dimorphum* and *C.nanhuaense* with *C.gloeosporioides*. Thus, we propose that *C.citrulli*, *C.juglandicola*, and *C.peakense* be considered as synonyms of *C.gloeosporioides*. Further genome-wide data studies are needed to clarify the species boundaries in this large clade (Liu et al. 2015).

## Supplementary Material

XML Treatment for Colletotrichum cordae

XML Treatment for Colletotrichum fioriniae

XML Treatment for Colletotrichum gloeosporioides

XML Treatment for Colletotrichum guangyuanense

XML Treatment for Colletotrichum juglandium

XML Treatment for Colletotrichum karsti

XML Treatment for Colletotrichum mengyinense

XML Treatment for Colletotrichum siamense

## ﻿Additional information

### Author ORCIDs

Yixuan Li https://orcid.org/0009-0007-6862-9605

Lu Lin https://orcid.org/0000-0003-3456-3760

Xinlei Fan https://orcid.org/0000-0002-4946-4442

### Data availability

All of the data that support the findings of this study are available in the main text or Supplementary Information.

## Supplementary materials

Supplementary material 1 Strains of the *Colletotrichum* species with details of host, location and GenBank accessions of the sequences Data type docx https://binary.pensoft.net/file/1127067This dataset is made available under the Open Database License (http://opendatacommons.org/licenses/odbl/1.0/). The Open Database License (ODbL) is a license agreement intended to allow users to freely share, modify, and use this Dataset while maintaining this same freedom for others, provided that the original source and author(s) are credited.Yixuan Li, Lu Lin, Jing Cao, Mingxu Gan, Xinlei Fan

Supplementary material 2 The statistics of ML trees in this study Data type docx https://binary.pensoft.net/file/1127068This dataset is made available under the Open Database License (http://opendatacommons.org/licenses/odbl/1.0/). The Open Database License (ODbL) is a license agreement intended to allow users to freely share, modify, and use this Dataset while maintaining this same freedom for others, provided that the original source and author(s) are credited.Yixuan Li, Lu Lin, Jing Cao, Mingxu Gan, Xinlei Fan
