# Supplementary material for: ﻿Three new species of Colletotrichum (Glomerellales, Glomerellaceae) associated with walnut (Juglansregia) anthracnose from China
Source: MycoKeys. 2024 Sep 3;108:147–67. doi: 10.3897/mycokeys.108.125382 (PMC11387834; doi:10.3897/mycokeys.108.125382)
Supplement: Supplementary material 1 — Strains of the Colletotrichum species with details of host, location and GenBank accessions of the sequences [file mycokeys-108-147-s001.docx]

**Table S1.** Strains of the *Colletotrichum* species with details of host, location and GenBank accessions of the sequences.

|  | **Strain** | **Group** | **Host** | **Origin** | **GenBank accession numbers** | | | | | |
| --- | --- | --- | --- | --- | --- | --- | --- | --- | --- | --- |
|  |  |  |  |  | **ITS** | ***gapdh*** | ***chs1*** | ***his3*** | ***act*** | ***tub2*** |
| *C. americanum* syn. *C. godetiae* | CBS 129917, CPC 16002 | Acutatum | *Schinus molle* | Mexico | JQ948441 | JQ948772 | JQ949102 | JQ949432 | JQ949762 | JQ950092 |
| *C. abscissum* | COAD 1877* | Acutatum | *Citrus sinensis* cv. | Brazil | KP843126 | KP843129 | KP843132 | KP843138 | KP843141 | KP843135 |
| *C. acerbum* | CBS 128530, ICMP 12921, PRJ 1199.3* | Acutatum | *Malus domestica* , bitter rot of fruit | New Zealand | JQ948459 | JQ948790 | JQ949120 | JQ949450 | JQ949780 | JQ950110 |
| *C. acutatum* | CBS 112996, ATCC 56816, STE-U 5292* | Acutatum | *Carica papaya* | Australia | JQ005776 | JQ948677 | JQ005797 | JQ005818 | JQ005839 | JQ005860 |
| *C. acutatum* | CBS 979.69 | Acutatum | *Coffea arabica* | Kenya | JQ948400 | JQ948731 | JQ949061 | JQ949391 | JQ949721 | JQ950051 |
| *C. aenigma* | ICMP 18608* | Gloeosporioides | *Perseaamericana* | Israel | JX010244 | JX010044 | JX009774 | — | JX009443 | JX010389 |
| *C. aenigma* | CFCC 55535 | Gloeosporioides | *Euonymus japonicus* | China | [OQ344719](https://www.ncbi.nlm.nih.gov/nuccore/JX010244) | [—](https://www.ncbi.nlm.nih.gov/nuccore/JX010044) | [OQ410548](https://www.ncbi.nlm.nih.gov/nuccore/JX009774) | OQ410632 | [—](https://www.ncbi.nlm.nih.gov/nuccore/JX009443) | [OQ410568](https://www.ncbi.nlm.nih.gov/nuccore/JX010389) |
| *C. aeschynomenes* | ICMP 17673*, ATCC 201874 | Gloeosporioides | *Aeschynomene virginica* | USA | JX010176 | JX009930 | JX009799 | — | JX009483 | JX010392 |
| *C. alatae* | CBS 304.67*, ICMP 17919 | Gloeosporioides | *Dioscorea alata* | India | JX010190 | JX009990 | JX009837 | — | JX009471 | JX010383 |
| *C. alienum* | ICMP 12071 * | Gloeosporioides | *Malus domestica* | New Zealand | JX010251 | JX010028 | JX009882 | — | JX009572 | JX010411 |
| *C. americanum* | RGM 3380, CCCT 23.24* | Acutatum | *Drimys winteri* | Chile | OR644563 | OR644970 | OR645022 | OR659700 | OR645076 | OR645128 |
| *C. analogum* | YMF1.06943 | Gloeosporioides | *Ageratina adenophora* | China | OK030860 | OK513663 | OK513559 | — | OK513599 | OK513629 |
| *C. analogum* | YMF1.07327 | Gloeosporioides | *Ageratina adenophora* | China | OK030861 | OK513664 | OK513560 | — | OK513600 | OK513630 |
| *C. analogum* | YMF1.07304 | Gloeosporioides | *Ageratina adenophora* | China | OK030862 | OK513665 | OK513561 | — | OK513601 | OK513631 |
| *C. annellatum* | CBS 129826, CH1* | Boninense | *Hevea indica* , leaf | Colombia | JQ005222 | JQ005309 | JQ005396 | JQ005483 | JQ005570 | JQ005656 |
| *C. aotearoa* | ICMP 18537* | Gloeosporioides | *Coprosma* sp. | New Zealand | JX010205 | JX010005 | JX009853 | — | JX009564 | JX010420 |
| *C. arboricola* | CBS 144795*, SAG 53350-12 | Acutatum | *Fuchsia magellanica* | Chile | MH817944 | MH817950 | — | — | MH817956 | MH817962 |
| *C. arecicola* | CGMCC 3.19667* | Gloeosporioides | *Areca catechu* | China | MK914635 | MK935455 | MK935541 | — | MK935374 | MK935498 |
| *C. artocarpicola* | MFLUCC 18-1167* | Gloeosporioides | *Artocarpus heterophyllus* | Thailand | MN415991 | MN435568 | MN435569 | — | MN435570 | MN435567 |
| *C. asianum* | ICMP 18580*, CBS 130418 | Gloeosporioides | *Coffea arabica* | Thailand | FJ972612 | JX010053 | JX009867 | — | JX009584 | JX010406 |
| *C. atlanticum* | LM898 | Gloeosporioides | *Etlingera elatior* | Brazil | — | MZ264093 | — | — | — | MZ270516 |
| *C. atlanticum* | LM938* | Gloeosporioides | *Etlingera elatior* | Brazil | — | MZ264107 | — | — | — | MZ270523 |
| *C. australe* | CBS 116478, HKUCC 2616* | Acutatum | *Trachycarpus fortunei* | South Africa | JQ948455 | JQ948786 | JQ949116 | JQ949446 | JQ949776 | JQ950106 |
| *C. australe* | CBS 131325, CPC 19820 | Acutatum | *Hakea* sp. | Australia | JQ948456 | JQ948787 | JQ949117 | JQ949447 | JQ949777 | JQ950107 |
| *C. australianum* | VPRI 43075* | Gloeosporioides | *Citrus sinensis* | Australia | MG572138 | MG572127 | MW091987 | — | MN442109 | MG572149 |
| *C. avicenniae* | MFLUCC 23-0289 | Gloeosporioides | leaf spots on *mangroves* | Thailand | OR856121 | OR886390 | OR886402 | — | OR886393 | OR886396 |
| *C. bambusicola* | LC8469, M288 | Bambusicola | *Petasites hybridus* | China | MZ595857 | MZ772869 | MZ799334 | MZ673877 | MZ664155 | MZ673978 |
| *C. bannaense* | CGMCC3.18887, YNML52* | Acutatum | *Hevea brasiliensis* | China | MG209638 | MG242006 | MG241996 | — | MG242002 | MG209660 |
| *C. bannaense* | YNWD31 | Acutatum | *Hevea brasiliensis* | China | MG209639 | MG242007 | MG241997 | — | MG242003 | MG209661 |
| *C. beeveri* | CBS 128527, ICMP 18594* | Boninense | *Brachyglottis repanda* | New Zealand | JQ005171 | JQ005258 | JQ005345 | JQ005432 | JQ005519 | JQ005605 |
| *C. beeveri* | NN004142 | Boninense | *Yucca* sp. | China | MZ595881 | MZ664082 | MZ799277 | MZ673901 | MZ664179 | — |
| *C. boninense* | CBS 123755, MAFF 305972* | Boninense | *Crinum asiaticum* var. *sinicum* | Japan | JQ005153 | JQ005240 | JQ005327 | JQ005414 | JQ005501 | JQ005588 |
| *C. brasiliense* | CBS 128501, ICMP 18607, PAS12* | Boninense | *Passiflora edulis ,* fruit anthracnose | Brazil | JQ005235 | JQ005322 | JQ005409 | JQ005496 | JQ005583 | JQ005669 |
| *C. brasiliense* | CBS 128528, ICMP 18606, PAS10 | Boninense | *Passiflora edulis* , fruit | Brazil | JQ005234 | JQ005321 | JQ005408 | JQ005495 | JQ005582 | JQ005668 |
| *C. brassicicola* | CBS 101059, LYN 16331* | Boninense | *Brassica oleracea var. gemmifera* , leaf spot | New Zealand | JQ005172 | JQ005259 | JQ005346 | JQ005433 | JQ005520 | JQ005606 |
| *C. brisbanense* | CBS 292.67, DPI 11711* | Acutatum | *Capsicum annuum* | Australia | JQ948291 | JQ948621 | JQ948952 | JQ949282 | JQ949612 | JQ949942 |
| *C. bromeliacearum* | LC13855, LC0951-2 | Boninense | *Bromeliaceae* | China | MZ595834 | MZ664079 | MZ799269 | MZ673845 | MZ664132 | OK360931 |
| *C. bromeliacearum* | LC13856, LC0951-3 | Boninense | *Bromeliaceae* | China | MZ595835 | MZ664080 | MZ799270 | MZ673846 | MZ664133 | OK360932 |
| *C. bromeliacearum* | LC0951* | Boninense | *Bromeliad* | China | MZ595832 | MZ664077 | MZ799267 | MZ673843 | MZ664130 | MZ673956 |
| *C. bromeliacearum* | LC13854, LC0951-1 | Boninense | *Bromeliad* | China | MZ595833 | MZ664078 | MZ799268 | MZ673844 | MZ664131 | OK360930 |
| *C. cairnsense* | BRIP 63642* | Acutatum | *Capsicum annuum* | Australia | KU923672 | KU923704 | KU923710 | KU923722 | KU923716 | KU923688 |
| *C. camelliae* | CGMCC 3.14925, LC1364* | Gloeosporioides | *Camellia sinensis* | China | KJ955081 | KJ954782 | MZ799255 | MZ673847 | KJ954363 | KJ955230 |
| *C. camelliae-japonicae* | CGMCC 3.18118*, LC6416 | Boninense | *Camellia japonica* | China | KX853165 | KX893584 | MZ799271 | MZ673859 | KX893576 | KX893580 |
| *C. cangyuanense* | YMF1.05001* | Gloeosporioides | *Ageratina adenophora* | China | OK030864 | OK513667 | OK513563 | — | OK513603 | OK513633 |
| *C. cangyuanense* | YMF1.04998 | Gloeosporioides | *Ageratina adenophora* | China | OK030865 | OK513668 | OK513564 | — | OK513604 | OK513634 |
| *C. carthami* | SAPA 100011* | Acutatum | *Carthamus tinctorium* | Japan | AB696998 | — | — | — | — | AB696992 |
| *C. castaneae* | GUCC 21268.4 | Gloeosporioides | spots on leaves of *Castanea mollissima* | China | OP722991 | OP737973 | OP715778 | — | OP715812 | OP720868 |
| *C. catinaense* | CBS 142417*, CPC 27978 | Boninense | *Citrus reticulata* | Italy | KY856400 | KY856224 | KY856136 | KY856307 | KY855971 | KY856482 |
| *C. chamaedoreae* | LC13867, NN052884 | Boninense | *Chamaedorea erumpens* | China | MZ595889 | MZ664083 | MZ799273 | MZ673909 | MZ664187 | MZ674007 |
| *C. chamaedoreae* | LC13868, NN052885* | Boninense | *Chamaedorea erumpens* | China | MZ595890 | MZ664084 | MZ799274 | MZ673910 | MZ664188 | MZ674008 |
| *C. changpingense* | CGMCC 3.17582*, SA0016, MFLUCC 15-0022 | Gloeosporioides | *Rhizome of Fragaria* × *ananass* | China | KP683152 | MZ664048 | KP852449 | — | KP683093 | MZ673952 |
| *C. chiangmaiense* | MFLUCC 18-0945* | Gloeosporioides | *Magnolia garrettii* | Thailand | MW346499 | MW548592 | MW623653 | — | MW655578 | — |
| *C. chongqingense* | CS0612* | Boninense | *Camellia sinensis* | China | MG602060 | MG602022 | MT976117 | — | MT976107 | MG602044 |
| *C. chrysanthemi* | IMI 364540, CPC 18930 | Acutatum | *Chrysanthemum coronarium* , leaf spot | China | JQ948273 | JQ948603 | JQ948934 | JQ949264 | JQ949594 | JQ949924 |
| *C. chrysophilum* | URM 7368, CMM 4268* | Gloeosporioides | *Musa* sp. | Brazil | KX094252 | KX094183 | KX094083 | — | KX093982 | KX094285 |
| *C. cigarro* | ICMP 18539* | Gloeosporioides | *Olea europaea* | Australia | JX010230 | JX009966 | JX009800 | MZ673837 | JX009523 | JX010434 |
| *C. citricola* | ZJUC34, CBS 134228, CGMCC 3.15227* | Boninense | *Citrus unshiu* | China | KC293576 | KC293736 | — | — | KC293616 | KC293656 |
| *C. citrulli* | CAASZT54 | Gloeosporioides | *Citrulluslanatus* | China | MZ475134 | OL456686 | OL901154 | — | OL449284 | OL456645 |
| *C. citrulli* | CAASZT52,CGMCC3.20769* | Gloeosporioides | *Citrulluslanatus* | China | [MZ475133](https://www.ncbi.nlm.nih.gov/nuccore/MZ475134) | [OL456685](https://www.ncbi.nlm.nih.gov/nuccore/OL456686) | [OL901153](https://www.ncbi.nlm.nih.gov/nuccore/OL901154) | — | [OL449283](https://www.ncbi.nlm.nih.gov/nuccore/OL449284) | [OL456644](https://www.ncbi.nlm.nih.gov/nuccore/OL456645) |
| *C. clidemiae* | ICMP 18658* | Gloeosporioides | *Clidemia hirta* | USA, Hawaii | JX010265 | JX009989 | JX009877 | — | JX009537 | JX010438 |
| *C. cliviigenum* | CBS 146825, CPC 38800* | Boninense | *Clivia sp* | South Africa | MZ064415 | MZ078178 | MZ078161 | MZ078180 | MZ078143 | MZ078260 |
| *C. cobbittiense* | BRIP 66219* | Gloeosporioides | *Cordyline stricta × C. australis* | Australia | MH087016 | MH094133 | MH094135 | MH094136 | MH094134 | MH094137 |
| *C. coffeae-arabicae* | PPDU26B | Gloeosporioides | *Coffea arabica* | Saudi Arabia | OR048779 | OR050760 | OR050742 | — | OR050690 | OR050787 |
| *C. coffeae-arabicae* | PPDU29F | Gloeosporioides | *Coffea arabica* | Saudi Arabia | OR048768 | OR050749 | OR050731 | — | OR050679 | OR050776 |
| *C. coffeae-arabicae* | PPDU32A | Gloeosporioides | *Coffea arabica* | Saudi Arabia | OR048764 | OR050745 | OR050727 | — | OR050675 | OR050772 |
| *C. colombiense* | CBS 129818* | Boninense | *Passiflora edulis* , leaf | Colombia | JQ005174 | JQ005261 | JQ005348 | JQ005435 | JQ005522 | JQ005608 |
| *C. condaoense* | CBS 134299* | Boninense | *Ipomoea pes-caprae* | Vietnam | MH229914 | MH229920 | MH229926 | MH229927 | — | MH229923 |
| *C. conoides* | CGMCC 3.17615, CAUG17, LC6226* | Gloeosporioides | *Chili pepper* | China | KP890168 | KP890162 | KP890156 | — | KP890144 | KP890174 |
| *C. constrictum* | CBS 128504, ICMP 12941* | Boninense | *Citrus limon*, fruit rot | New Zealand | JQ005238 | JQ005325 | JQ005412 | JQ005499 | JQ005586 | JQ005672 |
| *C. constrictum* | CBS 128503, ICMP 12936 | Boninense | *Solanum betaceum* , fruit rot | New Zealand | JQ005237 | JQ005324 | JQ005411 | JQ005498 | JQ005585 | JQ005671 |
| ***C. cordea*** | CFCC 70160 | **Gloeosporioides** | ***Juglans regia*** | **China** | **PP397135** | **PP425086** | **PP425045** | **—** | **PP425002** | **PP425127** |
| ***C. cordea*** | CFCC 59618* | **Gloeosporioides** | ***Juglans regia*** | **China** | **PP397136** | **PP425087** | **PP425046** | **—** | **PP425003** | **PP425128** |
| *C. cordylinicola* | MFLUCC 090551*, ICMP 18579 | Gloeosporioides | *Cordyline fruticosa* | Thailand | JX010226 | JX009975 | JX009864 | — | HM470235 | JX010440 |
| *C. cosmi* | CBS 853.73, PD 73/856* | Acutatum | *Cosmos* sp., seed | Netherlands | JQ948274 | JQ948604 | JQ948935 | JQ949265 | JQ949595 | JQ949925 |
| *C. costaricense* | CBS 330.75* | Acutatum | *Coffea arabica* cv. Typica, berry | Costa Rica | JQ948180 | JQ948510 | JQ948841 | JQ949171 | JQ949501 | JQ949831 |
| *C. costaricense* | CBS 211.78, IMI 309622 | Acutatum | *Coffea* sp., twig | Costa Rica | JQ948181 | JQ948511 | JQ948842 | JQ949172 | JQ949502 | JQ949832 |
| *C. cuscutae* | IMI 304802, CPC 18873* | Acutatum | *Cuscuta* sp. | Dominica | JQ948195 | JQ948525 | JQ948856 | JQ949186 | JQ949516 | JQ949846 |
| *C. cymbidiicola* | IMI 347923* | Boninense | *Cymbidium* sp., leaf lesion | Australia | JQ005166 | JQ005253 | JQ005340 | JQ005427 | JQ005514 | JQ005600 |
| *C. cymbidiicola* | CBS 128543, ICMP 18584 | Boninense | *Cymbidium* sp., leaf spot | New Zealand | JQ005167 | JQ005254 | JQ005341 | JQ005428 | JQ005515 | JQ005601 |
| *C. cymbidiicola* | CBS 123757, MAFF 306100 | Boninense | *Cymbidium* sp. | Japan | JQ005168 | JQ005255 | JQ005342 | JQ005429 | JQ005516 | JQ005602 |
| *C. dacrycarpi* | CBS 130241, ICMP 19107* | Boninense | *Dacrycarpus dacrydioides* , leaf endophyte | New Zealand | JQ005236 | JQ005323 | JQ005410 | JQ005497 | JQ005584 | JQ005670 |
| *C. dimorphum* | CGMCC 3.16083* YMF1.07309 | Gloeosporioides | *Ageratina adenophora* | China, Guizhou province, Pingtang county | OK030867 | OK513670 | OK513566 | — | OK513606 | OK513636 |
| *C. dimorphum* | YMF1.07303 | Gloeosporioides | *Ageratina adenophora* | China, Guizhou province, Pingtang county | OK030866 | OK513669 | OK513565 | — | OK513605 | OK513635 |
| *C. diversum* | LC11292, CQ775* | Boninense | *Philodendron selloum* | China | MZ595844 | MZ664081 | MZ799272 | MZ673864 | MZ664142 | MZ673965 |
| *C. doitungense* | MFLUCC 14-0128* | Boninense | *Dendrobium* sp. | Thailand | MF448524 | MH049480 | — | — | MH376385 | MH351277 |
| *C. dracaenigenum* | MFLUCC 19-0430* | Gloeosporioides | *Dracaena* sp. | Thailand | MN921250 | MT215577 | MT215575 | — | MT313686 | — |
| *C. endophyticum* | MFLUCC 13-0418, LC0324* | Gloeosporioides | *Pennisetum purpureum* | Thailand | KC633854 | KC832854 | MZ799261 | MZ673839 | KF306258 | MZ673954 |
| *C. endophyticum* syn. *fici-septicae* | CAUG28, YTJB1,MFLU 19-27708* | Gloeosporioides | *Capsicum* sp. | China | KP145441 | KP145413 | KP145385 | — | KP145329 | KP145469 |
| *C. eriobotryae* | GLMC 1935* | Acutatum | *Eriobotrya japonica* | Taiwan | MF772487 | MF795423 | MN191653 | MN191658 | MN191648 | MF795428 |
| *C. euonymi* | CFCC 55540 | Gloeosporioides | *Euonymus japonicus* | China | OQ344715 | — | OQ410544 | OQ410628 | — | OQ410564 |
| *C. euonymi* | CFCC 55537 | Gloeosporioides | *Euonymus japonicus* | China | OQ344716 | — | OQ410545 | OQ410629 | — | OQ410565 |
| *C. euonymi* | CFCC 55483 | Gloeosporioides | *Euonymus japonicus* | China | OQ344717 | — | OQ410546 | OQ410630 | — | OQ410566 |
| *C. euonymi* | CFCC 55542* | Gloeosporioides | *Euonymus japonicus* | China | OQ344718 | — | OQ410547 | OQ410631 | — | OQ410567 |
| *C. euonymicola* | CFCC 55486* | Gloeosporioides | *Euonymus japonicus* | China | OQ344723 | — | OQ410552 | OQ410636 | — | OQ410572 |
| *C. euonymicola* | CFCC 55539 | Gloeosporioides | *Euonymus japonicus* | China | OQ344724 | — | OQ410553 | OQ410637 | — | OQ410573 |
| *C. feijoicola* | CBS 144633* | Boninense | *Acca sellowiana* | Portugal | MK876413 | MK876475 | — | — | MK876466 | MK876507 |
| *C. feijoicola* | CPC 34245 | Boninense | *Acca sellowiana* | Portugal | MK876414 | MK876474 | MK876471 | MK876477 | MK876465 | MK876506 |
| *C. filicis* | CBS 101611* | Acutatum | *Fern* | Costa Rica | JQ948196 | JQ948526 | JQ948857 | JQ949187 | JQ949517 | JQ949847 |
| *C. fioriniae* | CBS 128517, ARSEF 10222, ERL 1257, EHS 58* | Acutatum | *Fiorinia externa* (elongate hemlock scale, insect) | USA | [JQ948292](https://www.ncbi.nlm.nih.gov/nuccore/KP145441) | [JQ948622](https://www.ncbi.nlm.nih.gov/nuccore/KP145413) | [JQ948953](https://www.ncbi.nlm.nih.gov/nuccore/KP145385) | JQ949283 | [JQ949613](https://www.ncbi.nlm.nih.gov/nuccore/KP145329) | [JQ949943](https://www.ncbi.nlm.nih.gov/nuccore/KP145469) |
| *C. fioriniae* | CBS 119293, MEP 1322 | Acutatum | *Vaccinium corymbosum* (blueberry), fruit | New Zealand | JQ948314 | JQ948644 | JQ948975 | JQ949305 | JQ949635 | JQ949965 |
| *C. fioriniae* | IMI 363003, CPC 18928 | Acutatum | *Camellia reticulata* | China | JQ948339 | JQ948669 | JQ949000 | JQ949330 | JQ949660 | JQ949990 |
| *C. fioriniae* | CBS 129948, RB128 | Acutatum | *Tulipa* sp. | UK | JQ948344 | JQ948674 | JQ949005 | JQ949335 | JQ949665 | JQ949995 |
| ***C. fioriniae*** | **CFCC 59932** | **Acutatum** | ***Juglans regia*** | **China** | **PP397112** | **—** | **PP425022** | **—** | **PP424986** | **PP425107** |
| *C. floscerae sp. nov.* | LM891 | Gloeosporioides | *Etlingera elatior* | Brazil | — | MZ264092 | — | — | — | MZ270515 |
| *C. floscerae sp. nov.* | LM916* | Gloeosporioides | *Etlingera elatior* | Brazil | — | MZ264099 | — | — | — | MZ270518 |
| *C. fructicola* | ICMP 18581*, CBS 130416 | Gloeosporioides | *Coffea arabica* | Thailand | JX010165 | JX010033 | JX009866 | — | FJ907426 | JX010405 |
| *C. fructivorum* | Coll1414, BPI 884103, CBS 133125* | Gloeosporioides | *Vaccinium macrocarpon* | Burlington | JX145145 | MZ664047 | MZ799259 | — | MZ664126 | JX145196 |
| *C. gardeniae* | GUCC 12049 | Gloeosporioides | *Gardenia jasminoides* | China | OP722995 | OP737963 | OP715766 | – | OP715801 | OP720858 |
| *C. gloeosporioides* | IMI 356878*, ICMP 17821, CBS 112999 | Gloeosporioides | *Citrus sinensis* | Italy | JX010152 | JX010056 | JX009818 | JQ005413 | JX009531 | JX010445 |
| *C. gloeosporioides* | CBS 273.51(*) = ICMP 19121 | Gloeosporioides | *Citruslimon* | Italy | JX010148 | JX010054 | JX009903 | — | JX009558 | — |
| *C. gloeosporioides* | DAR 76936 = ICMP 18738 | Gloeosporioides | *Caryaillinoinensis* | Australia | [JX010151](https://www.ncbi.nlm.nih.gov/nuccore/JX010148) | [JX009976](https://www.ncbi.nlm.nih.gov/nuccore/JX010054) | [JX009797](https://www.ncbi.nlm.nih.gov/nuccore/JX009903) | — | [JX009542](https://www.ncbi.nlm.nih.gov/nuccore/JX009558) | — |
| *C. gloeosporioides* | ICMP12939 | Gloeosporioides | *Citrus* sp. | New Zealand | [JX010149](https://www.ncbi.nlm.nih.gov/nuccore/JX010151) | [JX009931](https://www.ncbi.nlm.nih.gov/nuccore/JX009976) | [JX009747](https://www.ncbi.nlm.nih.gov/nuccore/JX009797) | — | [JX009462](https://www.ncbi.nlm.nih.gov/nuccore/JX009542) | — |
| *C. gloeosporioides* | CBS 119204 = ICMP 18678 | Gloeosporioides | *Puerarialobata* | USA | [JX010150](https://www.ncbi.nlm.nih.gov/nuccore/JX010149) | [JX010013](https://www.ncbi.nlm.nih.gov/nuccore/JX009931) | [JX009790](https://www.ncbi.nlm.nih.gov/nuccore/JX009747) | — | [JX009502](https://www.ncbi.nlm.nih.gov/nuccore/JX009462) | — |
| *C. gloeosporioides* | ICMP 12066 | Gloeosporioides | *Ficus* sp. | New Zealand | [JX010158](https://www.ncbi.nlm.nih.gov/nuccore/JX010150) | [JX009955](https://www.ncbi.nlm.nih.gov/nuccore/JX010013) | [JX009888](https://www.ncbi.nlm.nih.gov/nuccore/JX009790) | — | [JX009550](https://www.ncbi.nlm.nih.gov/nuccore/JX009502) | — |
| *C. gloeosporioides* | ICMP 18730 | Gloeosporioides | *Citrus* sp. | New Zealand | [JX010157](https://www.ncbi.nlm.nih.gov/nuccore/JX010158) | [JX009981](https://www.ncbi.nlm.nih.gov/nuccore/JX009955) | [JX009861](https://www.ncbi.nlm.nih.gov/nuccore/JX009888) | — | [JX009548](https://www.ncbi.nlm.nih.gov/nuccore/JX009550) | — |
| *C. gloeosporioides* | ICMP 12938 | Gloeosporioides | *Citrussinensis* | New Zealand | [JX010147](https://www.ncbi.nlm.nih.gov/nuccore/JX010157) | [JX009935](https://www.ncbi.nlm.nih.gov/nuccore/JX009981) | [JX009746](https://www.ncbi.nlm.nih.gov/nuccore/JX009861) | — | [JX009560](https://www.ncbi.nlm.nih.gov/nuccore/JX009548) | — |
| *C. gloeosporioides* | ICMP 18694 | Gloeosporioides | *Mangiferaindica* | South Africa | [JX010155](https://www.ncbi.nlm.nih.gov/nuccore/JX010147) | [JX009980](https://www.ncbi.nlm.nih.gov/nuccore/JX009935) | [JX009796](https://www.ncbi.nlm.nih.gov/nuccore/JX009746) | — | [JX009481](https://www.ncbi.nlm.nih.gov/nuccore/JX009560) | — |
| *C. gloeosporioides* | ICMP 18695 | Gloeosporioides | *Citrus* sp. | USA | [JX010153](https://www.ncbi.nlm.nih.gov/nuccore/JX010155) | [JX009979](https://www.ncbi.nlm.nih.gov/nuccore/JX009980) | [JX009779](https://www.ncbi.nlm.nih.gov/nuccore/JX009796) | — | [JX009494](https://www.ncbi.nlm.nih.gov/nuccore/JX009481) | — |
| *C. gloeosporioides* | ICMP 18697 | Gloeosporioides | *Vitisvinifera* | USA | [JX010154](https://www.ncbi.nlm.nih.gov/nuccore/JX010153) | [JX009987](https://www.ncbi.nlm.nih.gov/nuccore/JX009979) | [JX009780](https://www.ncbi.nlm.nih.gov/nuccore/JX009779) | — | [JX009557](https://www.ncbi.nlm.nih.gov/nuccore/JX009494) | — |
| *C. gloeosporioides* | CFCC 55544 | Gloeosporioides | *Euonymus japonicus* | China | [OQ344720](https://www.ncbi.nlm.nih.gov/nuccore/JX010154) | [—](https://www.ncbi.nlm.nih.gov/nuccore/JX009987) | [OQ410549](https://www.ncbi.nlm.nih.gov/nuccore/JX009780) | OQ410633 | [—](https://www.ncbi.nlm.nih.gov/nuccore/JX009557) | OQ410569 |
| *C. gloeosporioides* | CFCC 55545 | Gloeosporioides | *Euonymus japonicus* | China | OQ344721 | — | OQ410550 | OQ410634 | — | OQ410570 |
| *C. gloeosporioides* | CFCC 55547 | Gloeosporioides | *Euonymus japonicus* | China | OQ344722 | — | OQ410551 | OQ410635 | — | OQ410571 |
| ***C. gloeosporioides*** | **CFCC 70189** | **Gloeosporioides** | ***Juglans regia*** | **China** | **PP397123** | **PP425075** | **PP425033** | **—** | PP424993 | **PP425117** |
| ***C. gloeosporioides*** | **CFCC 59907** | **Gloeosporioides** | ***Juglans regia*** | **China** | **PP397113** | **—** | **PP425023** | **—** | **—** | **PP425108** |
| ***C. gloeosporioides*** | **CFCC 59940** | **Gloeosporioides** | ***Juglans regia*** | **China** | **PP397127** | **PP425079** | **PP425037** | **—** | **PP424995** | **PP425121** |
| ***C. gloeosporioides*** | **CFCC 59615** | **Gloeosporioides** | ***Juglans regia*** | **China** | **PP397120** | **PP425072** | **PP425030** | **—** | **—** | **PP425114** |
| ***C. gloeosporioides*** | **CFCC 59933** | **Gloeosporioides** | ***Juglans regia*** | **China** | **PP397124** | **PP425076** | **PP425034** | **—** | **—** | **PP425118** |
| ***C. gloeosporioides*** | **CFCC 59949** | **Gloeosporioides** | ***Juglans regia*** | **China** | **PP397129** | **PP425081** | **PP425039** | **—** | **PP424996** | **PP425123** |
| ***C. gloeosporioides*** | **CFCC 59925** | **Gloeosporioides** | ***Juglans regia*** | **China** | **PP397122** | **PP425074** | **PP425032** | **—** | **PP424992** | **PP425116** |
| ***C. gloeosporioides*** | **CFCC 59621** | **Gloeosporioides** | ***Juglans regia*** | **China** | **PP397116** | **PP425068** | **PP425026** | **—** | **PP424988** | **PP425111** |
| ***C. gloeosporioides*** | **CFCC 59913** | **Gloeosporioides** | ***Juglans regia*** | **China** | **PP397121** | **PP425073** | **PP425031** | **—** | **PP424991** | **PP425115** |
| ***C. gloeosporioides*** | **CFCC 59611** | **Gloeosporioides** | ***Juglans regia*** | **China** | **PP397119** | **PP425071** | **PP425029** | **—** | **PP424990** | **PP425113** |
| ***C. gloeosporioides*** | **CFCC 59938** | **Gloeosporioides** | ***Juglans regia*** | **China** | **PP397125** | **PP425077** | **PP425035** | **—** | **—** | **PP425119** |
| ***C. gloeosporioides*** | **CFCC 59634** | **Gloeosporioides** | ***Juglans regia*** | **China** | **PP397117** | **PP425069** | **PP425027** | **—** | **PP424989** | **PP425112** |
| ***C. gloeosporioides*** | **CFCC 59613** | **Gloeosporioides** | ***Juglans regia*** | **China** | **PP397115** | **PP425067** | **PP425025** | **—** | **—** | **PP425110** |
| ***C. gloeosporioides*** | **CFCC 59939** | **Gloeosporioides** | ***Juglans regia*** | **China** | **PP397126** | **PP425078** | **PP425036** | **—** | **PP424994** | **PP425120** |
| ***C. gloeosporioides*** | **CFCC 59633** | **Gloeosporioides** | ***Juglans regia*** | **China** | **PP397114** | **PP425066** | **PP425024** | **—** | **PP424987** | **PP425109** |
| ***C. gloeosporioides*** | **CFCC 59632** | **Gloeosporioides** | ***Juglans regia*** | **China** | **PP397118** | **PP425070** | **PP425028** | **—** | **—** | **—** |
| ***C. gloeosporioides*** | **CFCC 70190** | **Gloeosporioides** | ***Juglans regia*** | **China** | **PP397128** | **PP425080** | **PP425038** | **—** | **—** | **PP425122** |
| *C. godetiae* | CBS 133.44* | Acutatum | *Clarkia hybrida , cv.* Kelvon Glory, seed | Denmark | JQ948402 | JQ948733 | JQ949063 | JQ949393 | JQ949723 | JQ950053 |
| *C. godetiae* | CBS 126522, PD 88/472, BBA 70345 | Acutatum | *Prunus cerasus* , fruit, die-back | Netherlands | JQ948411 | JQ948742 | JQ949072 | JQ949402 | JQ949732 | JQ950062 |
| *C. gracile* | YMF:1.06939 | Gloeosporioides | *Ageratina adenophora* | China | OK030868 | OK513671 | OK513567 |  | OK513607 | OK513637 |
| *C. gracile* | YMF1.07329 | Gloeosporioides | *Ageratina adenophora* | China | OK030869 | OK513672 | OK513568 |  | OK513608 | OK513638 |
| *C. grevilleae* | CBS 132879, CPC 15481* | Gloeosporioides | *Grevillea* sp. | Italy | KC297078 | KC297010 | KC296987 | KC297056 | KC296941 | KC297102 |
| *C. grossum* | CGMCC3.17614, CAUG7, LC6227* | Gloeosporioides | *Chili pepper* | China | KP890165 | KP890159 | KP890153 | — | KP890141 | KP890171 |
| *C. guajavae* | IMI 350839, CPC 18893* | Acutatum | *Psidium guajava* , fruit | India | JQ948270 | JQ948600 | JQ948931 | JQ949261 | JQ949591 | JQ949921 |
| ***C. guangyuanense*** | **CFCC 59902*** | **Gloeosporioides** | ***Juglans regia*** | **China** | **PP397133** | **PP425084** | **PP425043** | **—** | **PP425000** | **PP425125** |
| ***C. guangyuanense*** | **CFCC 70249** | **Gloeosporioides** | ***Juglans regia*** | **China** | **PP397134** | **PP425085** | **PP425044** | **—** | **PP425001** | **PP425126** |
| ***C. guangyuanense*** | **CFCC 70161** | **Gloeosporioides** | ***Juglans regia*** | **China** | **PP397151** | **PP425101** | **PP425060** | **—** | **PP425016** | **PP425143** |
| ***C. guangyuanense*** | **CFCC 59912** | **Gloeosporioides** | ***Juglans regia*** | **China** | **PP397152** | **PP425102** | **PP425061** | **—** | **PP425017** | **PP425144** |
| *C. hebeiense* | MFLUCC13–0726* | Gloeosporioides | *Vitis vinifera* | China | KF156863 | KF377495 | KF289008 | — | KF377532 | KF288975 |
| *C. hederiicola* | MFLU 15-0689* | Gloeosporioides | *Hedera helix* | Italy | MN631384 | — | MN635794 | — | MN635795 | — |
| *C. helleniense* | CBS 142418, CPC 26844* | Gloeosporioides | *Poncirus trifoliata* | Greece, Arta | KY856446 | KY856270 | KY856186 | KY856361 | KY856019 | KY856528 |
| *C. henanense* | LC3030, CGMCC 3.17354, LF238* | Gloeosporioides | *Camellia sinensis* | China | KJ955109 | KJ954810 | MZ799256 | MZ673835 | KM023257 | KJ955257 |
| *C. hippeastri* | CBS 125376, CSSG1* | Boninense | *Hippeastrum vittatum* , leaf | China | JQ005231 | JQ005318 | JQ005405 | JQ005492 | JQ005579 | JQ005665 |
| *C. hippeastri* | CBS 241.78, IMI 304052 | Boninense | *Hippeastrum* sp. | Netherlands | JQ005232 | JQ005319 | JQ005406 | JQ005493 | JQ005580 | JQ005666 |
| *C. horii* | NBRC 7478*, ICMP 10492, MTCC 10841 | Gloeosporioides | *Diospyros kaki* | Japan | GQ329690 | GQ329681 | JX009752 | — | JX009438 | JX010450 |
| *C. hystricis* | CBS 142411, CPC 28153* | Gloeosporioides | *Citrus hystrix* | Italy, Catania | KY856450 | KY856274 | KY856190 | KY856365 | KY856023 | KY856532 |
| *C. indonesiense* | CBS 127551, CPC 14986* | Acutatum | *Eucalyptus* sp. | Indonesia | JQ948288 | JQ948618 | JQ948949 | JQ949279 | JQ949609 | JQ949939 |
| *C. javanense* | CBS 144963* | Acutatum | *Capsicum annuum* | Indonesia | MH846576 | MH846572 | MH846573 | MH846571 | MH846575 | MH846574 |
| *C. jiangxiense* | CGMCC 3.17361*, LC3266, LF488 | Gloeosporioides | *Camellia sinensis* | China | KJ955149 | KJ954850 | MZ799257 | — | KJ954427 | OK236389 |
| *C. jinpingense* | CCTCC AF2021056* | Gloeosporioides | *Hevea brasiliensis* | China | MZ165534 | MZ361085 | MZ352021 | — | MZ277632 | MZ277688 |
| *C. johnstonii* | CBS 128532, ICMP 12926, PRJ 1139.3* | Acutatum | *Solanum lycopersicum* , fruit rot | New Zealand | JQ948444 | JQ948774 | JQ949105 | JQ949435 | JQ949765 | JQ950095 |
| *C. johnstonii* | IMI 357027, CPC 18924, PRJ 1125.005 | Acutatum | *Citrus* sp. | New Zealand | JQ948443 | JQ948773 | JQ949104 | JQ949434 | JQ949764 | JQ950094 |
| *C. juglandicola* | CGMCC3.24312* | Gloeosporioides | *Juglansregia* | China | OQ263015 | OQ282973 | OR004793 | — | OQ282966 | OQ282980 |
| *C. juglandicola* | CGMCC3.24313 | Gloeosporioides | *Juglansregia* | China | [OQ263018](https://www.ncbi.nlm.nih.gov/nuccore/OQ263015) | [OQ282977](https://www.ncbi.nlm.nih.gov/nuccore/OQ282973) | [OR004797](https://www.ncbi.nlm.nih.gov/nuccore/OR004793) | — | [OQ282970](https://www.ncbi.nlm.nih.gov/nuccore/OQ282966) | [OQ282984](https://www.ncbi.nlm.nih.gov/nuccore/OQ282980) |
| ***C. juglandium*** | **CFCC 59974*** | **Gloeosporioides** | ***Juglans regia*** | **China** | **[PP397130](https://www.ncbi.nlm.nih.gov/nuccore/OQ263018)** | **[PP425082](https://www.ncbi.nlm.nih.gov/nuccore/OQ282977)** | **[PP425040](https://www.ncbi.nlm.nih.gov/nuccore/OR004797)** | **—** | **[PP424997](https://www.ncbi.nlm.nih.gov/nuccore/OQ282970)** | **[—](https://www.ncbi.nlm.nih.gov/nuccore/OQ282984)** |
| ***C. juglandium*** | **CFCC 70165** | **Gloeosporioides** | ***Juglans regia*** | **China** | **PP397131** | **PP425083** | **PP425041** | **—** | **PP424998** | **—** |
| *C. kahawae* | IMI 319418*, ICMP 17816 | Gloeosporioides | *Coffea arabica* | Kenya | JX010231 | JX010012 | JX009813 | MZ673838 | JX009452 | JX010444 |
| *C. karsti* | CBS 132134, CORCG6* | Boninense | *Vanda sp* | China | HM585409 | HM585391 | HM582023 | — | HM581995 | HM585428 |
| *C. karsti* | CBS 129833 | Boninense | *Musa sp.* | Mexico | JQ005175 | JQ005262 | JQ005349 | JQ005436 | JQ005523 | JQ005609 |
| *C. karsti* | CBS 129829 | Boninense | *Gossypium hirsutum* | Germany | JQ005189 | JQ005276 | JQ005363 | JQ005450 | JQ005537 | JQ005623 |
| *C. karsti* | CBS 106.91 | Boninense | *Carica papaya , fruit spots* | Brazil | JQ005220 | JQ005307 | JQ005394 | JQ005481 | JQ005568 | JQ005654 |
| *C. karsti* | CBS 110779 | Boninense | *Eucalyptus grandis* | South Africa | JQ005198 | JQ005285 | JQ005372 | JQ005459 | JQ005546 | JQ005632 |
| ***C. karsti*** | **CFCC 59901** | **Boninense** | ***Juglans regia*** | **China** | **PP397132** | **—** | **PP425042** | **PP425149** | **PP424999** | **PP425124** |
| *C. karstii* syn. *C. wuxuhaiense* | YMF1.04951 | Boninense | *Potamogeton crispus* | China | OL842173 | OL981268 | OL981294 | — | OL981242 | OL981228 |
| *C. karstii* syn. *C. wuxuhaiense* | F34 | Boninense | *Potamogeton crispus* | China | OL842175 | OL981270 | OL981296 | — | OL981244 | OL981230 |
| *C. kinghornii* | CBS 198.35* | Acutatum | *Phormium* sp. | UK | JQ948454 | JQ948784 | JQ949115 | JQ949445 | JQ949775 | JQ950105 |
| *C. kniphofiae* | CBS 143496* | Acutatum | *Kniphofia uvaria* | UK | MH107884 | MH107998 | MH107990 | — | MH107975 | MH108037 |
| *C. kunmingense* | GUCC 12053* | Gloeosporioides | Ophiopogon japonicus | China | OP722975 | OP737965 | OP715769 | — | OP715804 | OP720861 |
| *C. laticiphilum* | CBS 112989, IMI 383015, STE-U 5303* | Acutatum | *Hevea brasiliensis* | India | JQ948289 | JQ948619 | JQ948950 | JQ949280 | JQ949610 | JQ949940 |
| *C. laticiphilum* | CBS 129827, CH2 | Acutatum | *Hevea brasiliensis* | Colombia | JQ948290 | JQ948620 | JQ948951 | JQ949281 | JQ949611 | JQ949941 |
| *C. lauri* | MFLUCC:17-0205*, IT2505_1a | Acutatum | *Laurus nobilis* | Italy | KY514347 | KY514344 | KY514341 | — | KY514338 | KY514350 |
| *C. laurosilvaticum* | RGM 3086, CCCT 23.06 | Boninense | *Pitavia punctata* | Chile | OR644581 | OR644988 | OR645041 | OR659719 | OR645094 | OR645146 |
| *C. laurosilvaticum* | RGM 3406, CCCT 23.11* | Boninense | *Laurelia sempervirens* | Chile | OR644582 | OR644989 | OR645042 | OR659720 | OR645095 | OR645147 |
| *C. ledongense* | CGMCC3.18888* | Gloeosporioides | *Quercuspalustris* | China | MG242008 | MG242016 | MG242018 | — | MG242014 | MG242010 |
| *C. ligustri* | GUCC 12111 | Gloeosporioides | *Ilex chinensis* | China | OP722988 | OP737968 | OP715773 | — | OP740216 | OP720864 |
| *C. limetticola* | CBS 114.14* | Acutatum | *Citrus aurantifolia* , young twig | USA, Florida | JQ948193 | JQ948523 | JQ948854 | JQ949184 | JQ949514 | JQ949844 |
| *C. limonicola* | CBS 142409, CPC 27861 | Boninense | *Citrus limon* | Malta, Gozo | KY856471 | KY856295 | KY856212 | KY856387 | KY856044 | KY856553 |
| *C. limonicola* | CBS 142410*, CPC 31141 | Boninense | *Citrus limon* | Malta, Gozo | KY856472 | KY856296 | KY856213 | KY856388 | KY856045 | KY856554 |
| *C. lumnitzerae* | MFLUCC 23-0291 | Gloeosporioides | *leaf spots on* *mangroves* | Thailand | OR856122 | OR886391 | OR886403 | — | OR886394 | OR886397 |
| *C. lupini* | CBS 109225, BBA 70884* | Acutatum | *Lupinus albus* | Ukraine | JQ948155 | JQ948485 | JQ948816 | JQ949146 | JQ949476 | JQ949806 |
| *C. makassarense* | CBS 143664* | Gloeosporioides | *Capsicum annuum* | Indonesia | MH728812 | MH728820 | MH805850 | — | MH781480 | MH846563 |
| *C. melonis* | CBS 159.84* | Acutatum | *Cucumis melo* | Brazil | JQ948194 | JQ948524 | JQ948855 | JQ949185 | JQ949515 | JQ949845 |
| *C. mengyinense* | SAUCC0702* | Gloeosporioides | *Rosachinensis* | China | MW786742 | MW846240 | MW883686 | — | MW883695 | MW888970 |
| ***C. mengyinense*** | **CFCC 59923** | **Gloeosporioides** | ***Juglans regia*** | **China** | **[PP397144](https://www.ncbi.nlm.nih.gov/nuccore/MW786742)** | **[PP425094](https://www.ncbi.nlm.nih.gov/nuccore/MW846240)** | **[PP425053](https://www.ncbi.nlm.nih.gov/nuccore/MW883686)** | **—** | **[PP425011](https://www.ncbi.nlm.nih.gov/nuccore/MW883695)** | **[PP425136](https://www.ncbi.nlm.nih.gov/nuccore/MW888970)** |
| ***C. mengyinense*** | **CFCC 59935** | **Gloeosporioides** | ***Juglans regia*** | **China** | **PP397146** | **PP425096** | **PP425055** | **—** | **PP425012** | **PP425138** |
| ***C. mengyinense*** | **CFCC 59604** | **Gloeosporioides** | ***Juglans regia*** | **China** | **PP397143** | **PP425093** | **PP425052** | **—** | **PP425010** | **PP425135** |
| ***C. mengyinense*** | **CFCC 59950** | **Gloeosporioides** | ***Juglans regia*** | **China** | **PP397150** | **PP425100** | **PP425059** | **—** | **PP425015** | **PP425142** |
| ***C. mengyinense*** | **CFCC 59910** | **Gloeosporioides** | ***Juglans regia*** | **China** | **PP397142** | **PP425092** | **PP425051** | **—** | **PP425009** | **PP425134** |
| ***C. mengyinense*** | **CFCC 59903** | **Gloeosporioides** | ***Juglans regia*** | **China** | **PP397137** | **—** | **PP425047** | **—** | **PP425004** | **PP425129** |
| ***C. mengyinense*** | **CFCC 59608** | **Gloeosporioides** | ***Juglans regia*** | **China** | **PP397141** | **PP425091** | **PP425050** | **—** | **PP425008** | **PP425133** |
| ***C. mengyinense*** | **CFCC 59944** | **Gloeosporioides** | ***Juglans regia*** | **China** | **PP397148** | **PP425098** | **PP425057** | **—** | **PP425013** | **PP425140** |
| ***C. mengyinense*** | **CFCC 59908** | **Gloeosporioides** | ***Juglans regia*** | **China** | **PP397138** | **PP425088** | **PP425048** | **—** | **PP425005** | **PP425130** |
| ***C. mengyinense*** | **CFCC 59943** | **Gloeosporioides** | ***Juglans regia*** | **China** | **PP397147** | **PP425097** | **PP425056** | **—** | **—** | **PP425139** |
| ***C. mengyinense*** | **CFCC 59605** | **Gloeosporioides** | ***Juglans regia*** | **China** | **PP397140** | **PP425090** | **PP425049** | **—** | **PP425007** | **PP425132** |
| ***C. mengyinense*** | **CFCC 59945** | **Gloeosporioides** | ***Juglans regia*** | **China** | **PP397149** | **PP425099** | **PP425058** | **—** | **PP425014** | **PP425141** |
| ***C. mengyinense*** | **CFCC 70187** | **Gloeosporioides** | ***Juglans regia*** | **China** | **PP397145** | **PP425095** | **PP425054** | **—** | **—** | **PP425137** |
| ***C. mengyinense*** | **CFCC 59614** | **Gloeosporioides** | ***Juglans regia*** | **China** | **PP397139** | **PP425089** | **—** | **—** | PP425006 | **PP425131** |
| *C. miaoliense* | NTUCC 20-001-1* | Acutatum | *Fragaria* × *ananassa* | Taiwan | MK908419 | MK908470 | MK908522 | — | MK908573 | MK908624 |
| *C. musae* | CBS 116870*, ICMP 19119, MTCC 11349 | Gloeosporioides | *Musa* sp. | USA | JX010146 | JX010050 | JX009896 | — | JX009433 | HQ596280 |
| *C. murrayae* | GZAAS5.09506 | Gloeosporioides | *Murraya* sp. | China | JQ247633 | JQ247609 | — | — | JQ247657 | JQ247644 |
| *C. nanhuaensis* | CGMCC 3.18962* | Gloeosporioides | *Ageratinaadenophora* | China | OK030870 | OK513673 | OK513569 | — | OK513609 | OK513639 |
| *C. nanhuaensis* | YMF1.04990 | Gloeosporioides | *Ageratinaadenophora* | China | [OK030871](https://www.ncbi.nlm.nih.gov/nuccore/OK030870) | [OK513674](https://www.ncbi.nlm.nih.gov/nuccore/OK513673) | [OK513570](https://www.ncbi.nlm.nih.gov/nuccore/OK513569) | — | [OK513610](https://www.ncbi.nlm.nih.gov/nuccore/OK513609) | [OK513640](https://www.ncbi.nlm.nih.gov/nuccore/OK513639) |
| *C. nanjingense* | CFCC 58939 | Gloeosporioides | *J. mesnyi* | China | OQ456154 | OQ507143 | OQ507140 | — | OQ507150 | OQ507146 |
| *C. nanjingense* | CFCC 58940* | Gloeosporioides | *J. mesnyi* | China | OQ456155 | OQ507144 | OQ507141 | — | OQ507149 | OQ507147 |
| *C. novae-zelandiae* | CBS 128505, ICMP 12944* | Boninense | *Capsicum annuum* , fruit rot | New Zealand | JQ005228 | JQ005315 | JQ005402 | JQ005489 | JQ005576 | JQ005662 |
| *C. novae-zelandiae* | CBS 130240, ICMP 12064 | Boninense | *Citrus* sp. (grapefruit) | New Zealand | JQ005229 | JQ005316 | JQ005403 | JQ005490 | JQ005577 | JQ005663 |
| *C. noveboracense* | AFKH109 | Gloeosporioides | *Malus domestica* | USA | MN646685 | MN640567 | — | — | MN640565 | MN640569 |
| *C. noveboracense* | AFK423 | Gloeosporioides | *Apple/McIntosh* | Ulster/NY | MN708219 | MN741085 | — | — | MN701181 | MN701194 |
| *C. noveboracense* | AFK220 | Gloeosporioides | *Apple/McIntosh* | Ulster/NY | MN625451 | MN689180 | — | — | MN622839 | MN622861 |
| *C. nullisetosum* | YMF1.06946 | Gloeosporioides | *Mangifera indica* | China | OK030872 | OK513675 | OK513571 |  | OK513611 | OK513641 |
| *C. nupharicola* | CBS 470.96*, ICMP 18187 | Gloeosporioides | *Nuphar lutea sub* sp. *polysepala* | USA | JX010187 | JX009972 | JX009835 | — | JX009437 | JX010398 |
| *C. nymphaeae* | ZJUC42, CBS 134234 | Acutatum | *Citrus aurantifolia* | China | KC293582 | KC293742 | KY856139 | KY856310 | KY855974 | KC293662 |
| *C. nymphaeae* | CBS 515.78* | Acutatum | *Nymphaea alba* , leaf spot | Netherlands | JQ948197 | JQ948527 | JQ948858 | JQ949188 | JQ949518 | JQ949848 |
| *C. nymphaeae* | CBS 516.78, IAM 14670 | Acutatum | *Nuphar luteum* , leaf spot | Netherlands | JQ948198 | JQ948528 | JQ948859 | JQ949189 | JQ949519 | JQ949849 |
| *C. nymphaeae,* syn. *C. citri* | ZJUC41, CBS 134233, CGMCC 3.15228* | Acutatum | *Citrus aurantifolia* | China | KC293581 | KC293741 | KY856138 | KY856309 | KY855973 | KC293661 |
| *C. oblongisporum* | YMF1.06938 | Gloeosporioides | *Ageratina adenophora* | China | OK030874 | OK513677 | OK513573 | — | — | OK513643 |
| *C. oblongisporum* | YMF1.07326 | Gloeosporioides | *Ageratina adenophora* | China | OK030875 | OK513678 | OK513574 | — | — | OK513644 |
| *C. oncidii* | CBS 130242 | Boninense | *Oncidium* sp. | Germany | JQ005170 | JQ005257 | JQ005344 | JQ005431 | JQ005518 | JQ005604 |
| *C. oncidii* | CBS 129828* | Boninense | *Oncidium* sp., leaf | Germany | JQ005169 | JQ005256 | JQ005343 | JQ005430 | JQ005517 | JQ005603 |
| *C. orchidophilum* | CBS 632.80* | singleton | *Dendrobium sp.* | USA | JQ948151 | JQ948481 | JQ948812 | JQ949142 | JQ949472 | JQ949802 |
| *C. palki* | RGM 3055, CCCT 23.04* | Boninense | *Cestrum parqui* | Chile | OR644584 | OR644991 | OR645044 | OR659722 | OR645097 | OR645149 |
| *C. paranaense* | CBS 134729* | Acutatum | *Malus domestica* | Paraná | KC204992 | KC205026 | KC205043 | KC205004 | KC205077 | KC205060 |
| *C. parsonsiae* | CBS 128525, ICMP 18590* | Boninense | *Parsonsia capsularis*, leaf endophyte | New Zealand | JQ005233 | JQ005320 | JQ005407 | JQ005494 | JQ005581 | JQ005667 |
| *C. paxtonii* | IMI 165753, CPC 18868* | Acutatum | *Musa* sp. | Saint Lucia | JQ948285 | JQ948615 | JQ948946 | JQ949276 | JQ949606 | JQ949936 |
| *C. paxtonii* | CBS 502.97, LARS 58 | Acutatum | *Musa nana* | West Indies | JQ948286 | JQ948616 | JQ948947 | JQ949277 | JQ949607 | JQ949937 |
| *C. peakense* | CGMCC3.24308* | Gloeosporioides | *Juglans regia* | China | OQ263017 | OQ282975 | OR004795 | — | OQ282968 | OQ282982 |
| *C. peakense* | CGMCC3.24307 | Gloeosporioides | *Juglans regia* | China | [OQ263016](https://www.ncbi.nlm.nih.gov/nuccore/OQ263017) | [OQ282974](https://www.ncbi.nlm.nih.gov/nuccore/OQ282975) | [OR004794](https://www.ncbi.nlm.nih.gov/nuccore/OR004795) | — | [OQ282967](https://www.ncbi.nlm.nih.gov/nuccore/OQ282968) | [OQ282981](https://www.ncbi.nlm.nih.gov/nuccore/OQ282982) |
| *C. perseae* | CBS 141365*, GA100 | Gloeosporioides | *Avocado* | Israel | [KX620308](https://www.ncbi.nlm.nih.gov/nuccore/OQ263016) | [KX620242](https://www.ncbi.nlm.nih.gov/nuccore/OQ282974) | [MZ799260](https://www.ncbi.nlm.nih.gov/nuccore/OR004794) | — | [KX620145](https://www.ncbi.nlm.nih.gov/nuccore/OQ282967) | [KX620341](https://www.ncbi.nlm.nih.gov/nuccore/OQ282981) |
| *C. perseae* | GA319 | Gloeosporioides | *Avocado* | Israel | KX620322 | KX620256 | — | — | KX620159 | KX620355 |
| *C. perseae* | CBS 141366, GA272 | Gloeosporioides | *Avocado* | Israel | KX620321 | KX620255 | — | — | KX620158 | KX620354 |
| *C. perseicola* | RGM 3376, CCCT 23.27* | Acutatum | *Persea lingue* | Chile | OR644585 | OR644992 | OR645045 | OR659723 | OR645098 | OR645150 |
| *C. petchii* | CBS 378.94* | Boninense | *Dracaena marginata* , spotted leaves | Italy | JQ005223 | JQ005310 | JQ005397 | JQ005484 | JQ005571 | JQ005657 |
| *C. petchii* | CBS 118193, AR 3658 | Boninense | *Dracaena sanderana* , living leaves | China | JQ005227 | JQ005314 | JQ005401 | JQ005488 | JQ005575 | JQ005661 |
| *C. petchii* | CBS 125957, NB 145 | Boninense | *Dracaena* , leaf spots | Netherlands | JQ005226 | JQ005313 | JQ005400 | JQ005487 | JQ005574 | JQ005660 |
| *C. phormii* | CBS 118194, AR 3546* | Acutatum | *Phormium* sp. | Germany | JQ948446 | JQ948777 | JQ949107 | JQ949437 | JQ949767 | JQ950097 |
| *C. phormii* | CBS 199.35, DSM 1168 | Acutatum | *Phormium* sp. | UK | JQ948447 | JQ948778 | JQ949108 | JQ949438 | JQ949768 | JQ950098 |
| *C. phyllanthi* | CBS 175.67, MACS 271* | Boninense | *Phyllanthus acidus* | India | JQ005221 | JQ005308 | JQ005395 | JQ005482 | JQ005569 | JQ005655 |
| *C. polypodialium* | MFLUCC 22-0178 | Gloeosporioides | *Nephrolepis* sp. | Thailand | OP802361 | OP801720 | OP801702 | — | OP801685 | OP801739 |
| *C. proteae* | CBS 132882*, CPC 14859 | Gloeosporioides | *Protea* sp. | South Africa | KC297079 | KC297009 | KC296986 | KC297045 | KC296940 | KC297101 |
| *C. pseudotheobromicola* | MFLUCC 18-1602* | Gloeosporioides | *Prunusavium* | China | MH817395 | MH853675 | MH853678 | — | MH853681 | MH853684 |
| *C. psidii* | CBS 145.29*, ICMP 19120 | Gloeosporioides | *Psidium* sp. | Italy | [JX010219](https://www.ncbi.nlm.nih.gov/nuccore/MH817395) | [JX009967](https://www.ncbi.nlm.nih.gov/nuccore/MH853675) | [JX009901](https://www.ncbi.nlm.nih.gov/nuccore/MH853678) | — | [JX009515](https://www.ncbi.nlm.nih.gov/nuccore/MH853681) | [JX010443](https://www.ncbi.nlm.nih.gov/nuccore/MH853684) |
| *C. pyricola* | CBS 132893 | Acutatum | Unidentified | Tasmania | OR644586 | OR644993 | OR645046 | OR659724 | OR645099 | OR645151 |
| *C. pyricola* | CBS 128531, ICMP 12924, PRJ 977.1* | Acutatum | *Pyrus communis* , fruit rot | New Zealand | JQ948445 | JQ948776 | JQ949106 | JQ949436 | JQ949766 | JQ950096 |
| *C. queenslandicum* | ICMP 1778* | Gloeosporioides | *Carica papaya* | Australia | JX010276 | JX009934 | JX009899 | — | JX009447 | JX010414 |
| *C. rhexiae* | Coll1026, BPI 884112, CBS 133134* | Gloeosporioides | *Rhexia virginica* | Sussex | JX145128 | MZ664046 | MZ799258 | MZ673834 | MZ664127 | JX145179 |
| *C. rhombiforme* | CBS 129953, PT250, RB011* | Acutatum | *Olea europaea* | Portugal | JQ948457 | JQ948788 | JQ949118 | JQ949448 | JQ949778 | JQ950108 |
| *C. rhombiforme* | CBS 131322, DAOM 233253, C10, MS1L34 | Acutatum | *Vaccinium macrocarpum* | USA | JQ948458 | JQ948789 | JQ949119 | JQ949449 | JQ949779 | JQ950109 |
| *C. roseum* | CBS 145754* | Acutatum | *Lapageria rosea* | Chile | MK903611 | MK903603 | — | — | MK903604 | MK903607 |
| *C. salicis* | CBS 607.94* | Acutatum | *Salix* sp., leaf, spot | Netherlands | JQ948460 | JQ948791 | JQ949121 | JQ949451 | JQ949781 | JQ950111 |
| *C. salsolae* | ICMP 19051* | Gloeosporioides | *Salsola tragus* | Hungary | JX010242 | JX009916 | JX009863 | — | JX009562 | JX010403 |
| *C. saudianum* | PPDU38H* | Gloeosporioides | *Coffea arabica* | Saudi Arabia | OR048759 | — | OR050722 | — | OR050670 | OR050767 |
| *C. saudianum* | PPDU38F | Gloeosporioides | *Coffea arabica* | Saudi Arabia | OR048760 | — | OR050723 | — | OR050671 | OR050768 |
| *C. saudianum* | PPDU29B | Gloeosporioides | *Coffea arabica* | Saudi Arabia | OR048769 | OR050750 | OR050732 | — | OR050680 | OR050777 |
| *C. saudianum* | PPDU29A | Gloeosporioides | *Coffea arabica* | Saudi Arabia | OR048770 | OR050751 | OR050733 | — | OR050681 | OR050778 |
| *C. schimae* | LC13880, NN046984* | Acutatum | *Schima* sp. | China | MZ595885 | MZ664105 | MZ799347 | MZ673905 | MZ664183 | MZ674003 |
| *C. schimae* | LC13881, NN047247 | Acutatum | *Schima* sp. | China | MZ595887 | MZ664106 | MZ799348 | MZ673907 | MZ664185 | MZ674005 |
| *C. scovillei* | CBS 126529, PD 94/921-3, BBA 70349* | Acutatum | *Capsicum* sp. | Indonesia | JQ948267 | JQ948597 | JQ948928 | JQ949258 | JQ949588 | JQ949918 |
| *C. scovillei* | CBS 126530, PD 94/921-4 | Acutatum | *Capsicum* sp. | Indonesia | JQ948268 | JQ948598 | JQ948929 | JQ949259 | JQ949589 | JQ949919 |
| *C. scovillei* | CBS 120708, HKUCC 10893, Mj6 | Acutatum | *Capsicum annuum* | Thailand | JQ948269 | JQ948599 | JQ948930 | JQ949260 | JQ949590 | JQ949920 |
| *C. siamense* | ICMP 18578*, CBS 130417 | Gloeosporioides | *Coffea arabica* | Thailand | JX010171 | JX009924 | JX009865 | — | FJ907423 | JX010404 |
| *C. siamense* | ICMP 18118 | Gloeosporioides | *Commelina* sp. | Nigeria | JX010163 | JX009941 | JX009843 | — | JX009505 | JX010402 |
| *C. siamense* | BRIP 54270b, VPRI 43029, A10-43029 | Gloeosporioides | *Citrus australasica* | Australia | MK469995 | MK470013 | MW091971 | — | MK470085 | MK470049 |
| *C. siamense* | MFLUCC 18-1162 | Gloeosporioides | Unknown | Unknown | MN788676 | MN995328 | MN995335 | — | MN995334 | MN995329 |
| ***C. siamense*** | **CFCC 59625** | **Gloeosporioides** | ***Juglans regia*** | **China** | **PP397153** | **PP425103** | **PP425062** | **—** | **PP425018** | **PP425145** |
| ***C. siamense*** | **CFCC 59964** | **Gloeosporioides** | ***Juglans regia*** | **China** | **PP397154** | **PP425104** | **PP425063** | **—** | **PP425019** | **PP425146** |
| ***C. siamense*** | **CFCC 59601** | **Gloeosporioides** | ***Juglans regia*** | **China** | **PP397155** | **PP425105** | **PP425064** | **—** | **PP425020** | **PP425147** |
| ***C. siamense*** | **CFCC 59909** | **Gloeosporioides** | ***Juglans regia*** | **China** | **PP397156** | **PP425106** | **PP425065** | **—** | **PP425021** | **PP425148** |
| *C. siamense* syn. *C. endomangiferae* | CMM 3814a | Gloeosporioides | *Mangifera indica* | Brazil | KC702994 | KC702955 | KC598113 | — | KC702922 | KM404170 |
| *C. siamense* syn. *C. hymenocallidis* | CBS 116868 | Gloeosporioides | *Protea cynaroides* | Zimbabwe | KC566815 | KC566669 | KC566382 | — | KC566961 | KP703429 |
| *C. siamense* syn. *C. jasmini-sambac* | LLTA-01, MFLUCC 10–0277* | Gloeosporioides | *Jasmin sambac* | Vietnam | HM131511 | HM131497 | — | — | HM131507 | HM153768 |
| *C. siamense* syn. *C. menglaense* | YMF1.04960 | Gloeosporioides | air | China | MH023505 | MH023507 | MH023508 | — | MH023506 | — |
| *C. siamense* syn. *C. pandanicola* | MFLUCC 17-0571* | Gloeosporioides | *Pandanaceae* | Thailand | MG646967 | MG646934 | MG646931 | — | MG646938 | MG646926 |
| *C. siamense* syn. *C. parvisporum* | YMF1.06942 | Gloeosporioides | *Ageratina adenophora* | China | OK030876 | OK513679 | OK513575 | — | OK513613 | OK513645 |
| *C. siamense* syn. *C. rhizophorae* | MFLUCC 17-1927* | Gloeosporioides | *Rhizophora apiculata* | Thailand | OR828933 | OR840870 | OR840858 | — | OR840847 | OR840864 |
| *C. siamense* syn. *C. thailandica* | MFLUCC 17-1924* | Gloeosporioides | Rhizophora apiculata | Thailand | OR828935 | OR840872 | OR840860 | — | OR840849 | OR840866 |
| *C. simmondsii* | CBS 122122, BRIP 28519* | Acutatum | *Carica papaya*, fruit | Australia | JQ948276 | JQ948606 | JQ948937 | JQ949267 | JQ949597 | JQ949927 |
| *C. simmondsii* | CBS 295.67, DPI 16518 | Acutatum | *Fragaria* sp., fruit | Australia | JQ948278 | JQ948608 | JQ948939 | JQ949269 | JQ949599 | JQ949929 |
| *C. sloanei* | IMI 364297, CPC 18929* | Acutatum | *Theobroma cacao* | Malaysia | JQ948287 | JQ948617 | JQ948948 | JQ949278 | JQ949608 | JQ949938 |
| *C. sloanei* | MT27-4-b, BCRC FU31403 | Acutatum | *Mangifera indica* | Taiwan | MN809384 | MN820683 | MN820675 | MN820691 | MN820667 | MN810341 |
| *C. spicati* | YMF1.04942 | Boninense | *Myriophyllum spicatum* | China | OL842171 | OL981266 | OL981292 | — | OL981240 | OL981226 |
| *C. subhenanense* | YMF1.07324 | Gloeosporioides | *Ageratina adenophora* | China | OK030884 | OK513685 | OK513582 | — | OK513619 | OK513648 |
| *C. subsalicis* | LC13863, CQ1168* | Acutatum | *Populus alba* | China | MZ852849 | — | MZ799346 | MZ673836 | MZ664128 | MZ673953 |
| *C. syzygiicola* | DNCL021, MFLUCC 10-0624* | Gloeosporioides | *Syzygium samarangense* | Thailand | KF242094 | KF242156 | — | — | KF157801 | KF254880 |
| *C. tainanense* | CBS 143666* | Gloeosporioides | *Capsicum annuum* | Taiwan | MH728818 | MH728823 | MH805845 | — | MH781475 | MH846558 |
| *C. tamarilloi* | CBS 129814, T.A.6* | Acutatum | *Solanum betaceum*, fruit, anthracnose | Colombia | JQ948184 | JQ948514 | JQ948845 | JQ949175 | JQ949505 | JQ949835 |
| *C. tamarilloi* | CBS 129811, T.A.3 | Acutatum | *Solanum betaceum*, fruit, anthracnose | Colombia | JQ948185 | JQ948515 | JQ948846 | JQ949176 | JQ949506 | JQ949836 |
| *C. temperatum* | CBS 133122*, Coll883, BPI 884100 | Gloeosporioides | *Vaccinium macrocarpon* | Bronx | JX145159 | MZ664045 | MZ799254 | MZ673833 | MZ664125 | JX145211 |
| *C. tengchongense* | YMF1.04950 | Gloeosporioides | *Isoetessinensis* | China | OL842169 | OL981264 | OL981290 | — | OL981238 | — |
| *C. thasutense* | MFLU 22–0206 | Gloeosporioides | *Syngonium* sp. | Thailand | OP821902 | OP831282 | OP831281 | — | OP831280 | OP831283 |
| *C. theobromicola* | CBS 124945*, ICMP 18649 | Gloeosporioides | *Theobroma cacao* | Panama | [JX010294](https://www.ncbi.nlm.nih.gov/nuccore/OL842169) | [JX010006](https://www.ncbi.nlm.nih.gov/nuccore/OL981264) | [JX009869](https://www.ncbi.nlm.nih.gov/nuccore/OL981290) | — | [JX009444](https://www.ncbi.nlm.nih.gov/nuccore/OL981238) | JX010447 |
| *C. ti* | ICMP 4832* | Gloeosporioides | *Cordyline* sp. | New Zealand | JX010269 | JX009952 | JX009898 | — | JX009520 | JX010442 |
| *C. tomentosae* | ZHKUCC 21-0103 CGMCC 3.24128 | Gloeosporioides | *Citrusgrandis* cv. | China | OL708422 | OL855850 | OL855860 | — | OL855870 | OL855887 |
| *C. tomentosae* | ZHKU 21-0088 | Gloeosporioides | *Citrusgrandis* cv. | China | OL708419 | OL855856 | OL855866 | — | OL855873 | OL855888 |
| *C. tomentosae* | ZHKUCC 21-0104 | Gloeosporioides | *Citrusgrandis* cv. | China | ON303476 | ON315382 | ON315376 | — | ON315380 | ON315378 |
| *C. torulosum* | CBS 128544, ICMP 18586* | Boninense | *Solanum melongena* | New Zealand | JQ005164 | JQ005251 | JQ005338 | JQ005425 | JQ005512 | JQ005598 |
| *C. torulosum* | CBS 102667 | Boninense | *Passiflora edulis*, leaf blotch | New Zealand | JQ005165 | JQ005252 | JQ005339 | JQ005426 | JQ005513 | JQ005599 |
| *C. tropicale* | CBS 124949*, ICMP 18653, MTCC 11371 | Gloeosporioides | *Theobroma cacao* | Panama | JX010264 | JX010007 | JX009870 | MZ673832 | JX009489 | JX010407 |
| *C. viniferum* | GZAAS 5.08601*, yg1 | Gloeosporioides | *Vitis vinifera* cv. Shuijing | China | JN412804 | JN412798 | — | — | JN412795 | — |
| *C. vulgaris* | YMF1.04940 | Gloeosporioides | *Hippurisvulgaris* | China | OL842170 | OL981265 | OL981291 |  | OL981239 | N/A |
| *C. walleri* | CBS 125472, BMT(HL)19* | Acutatum | *Coffea* sp., leaf tissue | Vietnam | [JQ948275](https://www.ncbi.nlm.nih.gov/nuccore/OL842170) | [JQ948605](https://www.ncbi.nlm.nih.gov/nuccore/OL981265) | [JQ948936](https://www.ncbi.nlm.nih.gov/nuccore/OL981291) | JQ949266 | [JQ949596](https://www.ncbi.nlm.nih.gov/nuccore/OL981239) | JQ949926 |
| *C. wanningense* | CGMCC 3.18936* | Acutatum | *Hevea brasiliensis* | China | MG830462 | MG830318 | MG830302 | — | MG830270 | MG830286 |
| *C. wanningense* syn. *C. australisinense* | CGMCC3.18886, GX1655, LD1680* | Acutatum | *Hevea brasiliensis* | China | MG209623 | MG241962 | MG241981 | — | MG241947 | MG209645 |
| *C. watphraense* | MFLUCC 14-0123* | Boninense | *Dendrobium* sp. | Thailand | MF448523 | MH049479 | — | — | MH376384 | MH351276 |
| *C. wuxiense* | CGMCC 3.17894* | Gloeosporioides | *Camellia sinensis* | China | KU251591 | KU252045 | KU251939 | — | KU251672 | KU252200 |
| *C. xanthorrhoeae* | BRIP 45094*, ICMP 17903, CBS 127831 | Gloeosporioides | *Xanthorrhoea preissii* | Australia | JX010261 | JX009927 | JX009823 | — | JX009478 | JX010448 |
| *C. xishuangbannaense* | MFLUCC 19-0107* | Gloeosporioides | *Magnolia liliifera* | China | MW346469 | MW537586 | MW660832 | — | MW652294 | — |
| *C. yulongense* | CFCC 50818* | Gloeosporioides | *Vaccinium dunalianum* var. *urophyllum* | China | [MH751507](https://www.ncbi.nlm.nih.gov/nuccore/MW346469) | [MK108986](https://www.ncbi.nlm.nih.gov/nuccore/MW537586) | [MH793605](https://www.ncbi.nlm.nih.gov/nuccore/MW660832) | — | [MH777394](https://www.ncbi.nlm.nih.gov/nuccore/MW652294) | MK108987 |
| *C. yunanjiangensis* | CGMCC 3.18964* | Gloeosporioides | *Ageratinaadenophora* | China | OK030885 | OK513686 | OK513583 | — | OK513620 | OK513649 |
| *C. zingibericola* | LM937 | Gloeosporioides | *Etlingera elatior* | Brazil | — | MZ264106 | — | — | — | MZ270522 |
| *C. zingibericola* | LM942* | Gloeosporioides | *Etlingera elatior* | Brazil | — | MZ264104 | — | — | — | MZ270520 |

* Ex-type culture. Strains studied in this paper are in bold.
