# Supplementary material for: ﻿Three new species of Colletotrichum (Glomerellales, Glomerellaceae) associated with walnut (Juglansregia) anthracnose from China
Source: MycoKeys. 2024 Sep 3;108:147–67. doi: 10.3897/mycokeys.108.125382 (PMC11387834; doi:10.3897/mycokeys.108.125382)
Supplement: Supplementary material 2 — The statistics of ML trees in this study [file mycokeys-108-147-s002.docx]

**Table S2.** The statistics of ML trees in this study.

| **Analysis** | **Fig. number** | **Estimated base frequencies** | | | | **substitution rates** | | | | | | **Gamma distribution shape parameter α** |
| --- | --- | --- | --- | --- | --- | --- | --- | --- | --- | --- | --- | --- |
|  |  | **A** | **T** | **C** | **G** | **AC** | **AG** | **AT** | **CG** | **CT** | **GT** |  |
| *Colletotrichum*  *acutatum* complex | Fig. 2 | 0.224433 | 0.224736 | 0.310798 | 0.240033 | 1.290662 | 3.269972 | 1.449704 | 0.704839 | 6.920531 | 1.000000 | 0.415184 |
| *Colletotrichum boninense* complex | Fig. 3 | 0.222474 | 0.219401 | 0.312418 | 0.313330 | 1.150368 | 2.594356 | 0.907052 | 0.792623 | 4.519841 | 1.000000 | 0.299185 |
| *Colletotrichum gloeosporioides* complex | Fig. 4 | 0.231865 | 0.229308 | 0.299751 | 0.239076 | 0.893705 | 3.226617 | 1.093116 | 0.944504 | 4.217853 | 1.000000 | 0.537494 |
